# E3 ubiquitin ligase Listerin regulates macrophage cholesterol efflux and atherosclerosis by targeting ABCA1

**DOI:** 10.1172/JCI186509

**Published:** 2025-06-17

**Authors:** Lei Cao, Jie Zhang, Liwen Yu, Wei Yang, Wenqian Qi, Ruiqing Ren, Yapeng Liu, Yonghao Hou, Yu Cao, Qian Li, Xiaohong Wang, Zhengguo Zhang, Bo Li, Wenhai Sui, Yun Zhang, Chengjiang Gao, Cheng Zhang, Meng Zhang

**Affiliations:** 1 State Key Laboratory for Innovation and Transformation of Luobing Theory; Key Laboratory of Cardiovascular Remodeling and Function Research, Chinese Ministry of Education, Chinese National Health Commission and Chinese Academy of Medical Sciences, Department of Cardiology and; 2 Organ Procurement Organizations, Qilu Hospital of Shandong University, Jinan, Shandong, China.; 3 Department of Cardiology, Zibo Central Hospital, Zibo, Shandong, China.; 4 Cardiovascular Disease Research Center, Jinan Central Hospital, Shandong First Medical University, Jinan, Shandong, China.; 5 Key Laboratory of Infection and Immunity of Shandong Province, Department of Immunology, Cheeloo College of Medicine, Shandong University, Jinan, China.

**Keywords:** Cardiology, Metabolism, Atherosclerosis, Ubiquitin-proteosome system

## Abstract

Atherosclerosis arises from disrupted cholesterol metabolism, notably impaired macrophage cholesterol efflux leading to foam cell formation. Through single-cell and bulk RNA-Seq, we identified Listerin E3 ubiquitin protein ligase 1 (Listerin) as a regulator of macrophage cholesterol metabolism. Listerin expression increased during atherosclerosis progression in humans and rodents. Its deficiency suppressed cholesterol efflux, promoted foam cell formation, and exacerbated plaque features (macrophage infiltration, lipid deposition, necrotic cores) in macrophage-specific KO mice. Conversely, Listerin overexpression attenuated these atherosclerotic manifestations. Mechanistically, Listerin stabilizes ABCA1, a key cholesterol efflux mediator, by catalyzing K63-linked polyubiquitination at residues K1884/K1957, countering ESCRT-mediated lysosomal degradation of ABCA1 induced by oxidized LDL (oxLDL). ABCA1 agonist erythrodiol restored cholesterol efflux in Listerin-deficient macrophages, while KO of ABCA1 abolished Listerin’s effects in Tsuchiya human monocytic leukemia line (THP-1) cells. This study establishes Listerin as a protective factor in atherosclerosis via posttranslational stabilization of ABCA1, offering a potential therapeutic strategy targeting ABCA1 ubiquitination to enhance cholesterol efflux.

## Abstract 2

E3 ligase Listerin expression is positively correlated with atherosclerosis development in humans and mice, which is confirmed by single-cell and bulk RNA sequencing. In addition, Listerin specificity knockout in macrophages aggravates atherosclerosis progression, Conversely, Listerin overexpression improves atherosclerosis progression. Mechanistically, we demonstrated that Listerin regulates macrophage cholesterol efflux by blocking ABCA1 degradation via ESCRT lysosomal pathway for the first time.

## Graphical abstract

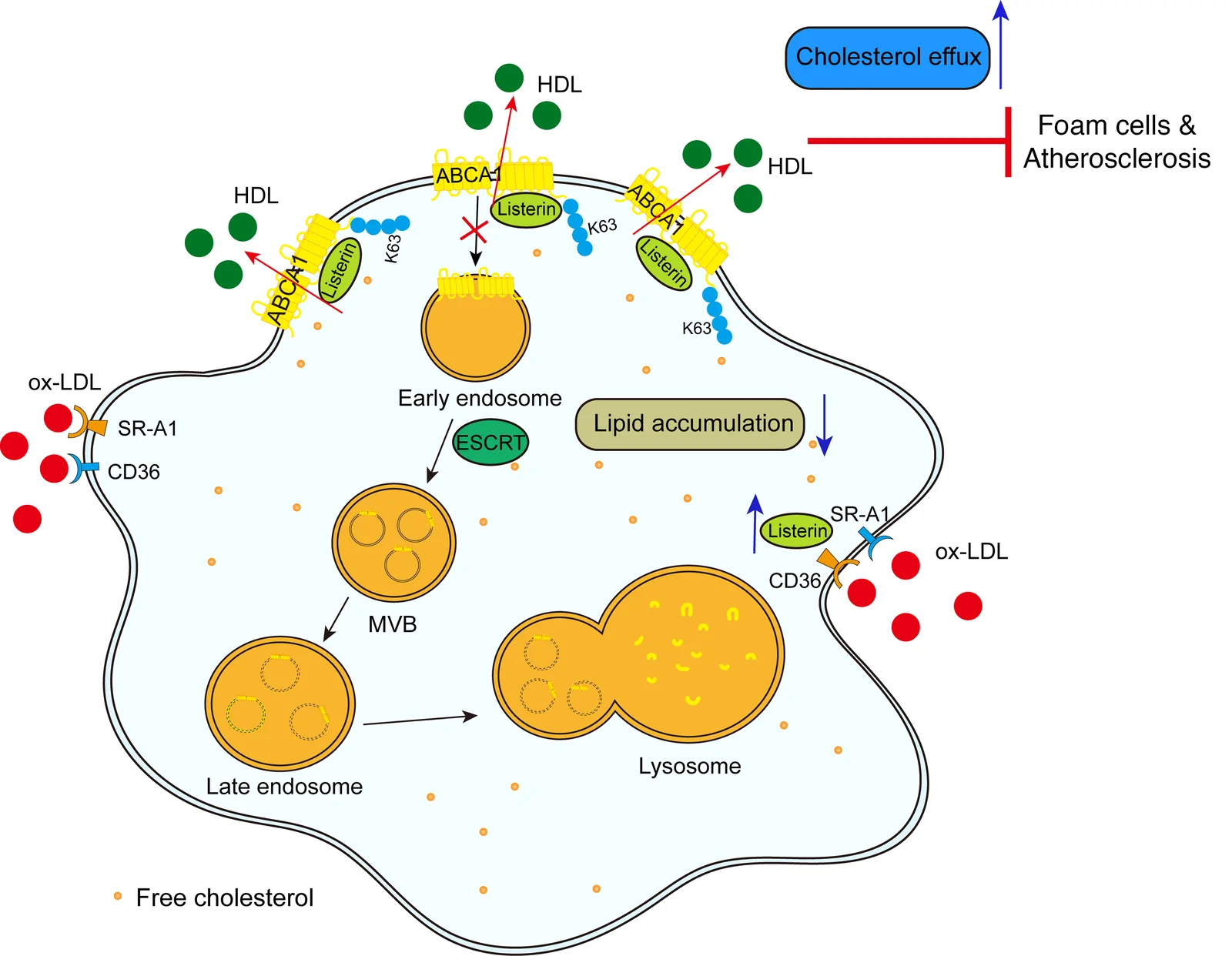

## Introduction

Atherosclerotic cardiovascular disease (ASCVD) is the leading cause of morbidity and mortality worldwide (1–4). Atherosclerosis is characterized by excessive lipid deposition in the intima of the arterial wall, and macrophages play key roles in this process (2, 5–7). Monocytes in the blood enter the subintima to differentiate into macrophages and phagocytose-modified lipoproteins (8–11). In persistent hyperlipidemia, macrophages become foam cells and constitute the core of the atherosclerotic plaque (4, 9, 12). Unstable plaques progress to plaque rupture and thrombosis, leading to acute myocardial ischemia and myocardial infarction (10). Although macrophage foam cell formation plays an indispensable role in atherosclerosis, the underlying molecular mechanisms have not been fully elucidated.

ATP-binding cassette transporter A1 (ABCA1) is an integral membrane protein with a molecular weight of 254 kDa and is expressed on the plasma and endosomal membranes of many tissues (13–17). ABCA1 promotes the efflux of plasma membrane-free cholesterol and phospholipids to apolipoprotein A-1 (ApoA1) and forms nascent HDL (18, 19). Numerous studies have shown that ABCA1 plays an essential role in reverse cholesterol transport (RCT) in macrophages by stimulating the efflux of cholesterol, thereby reducing foam cell formation and the progression of atherosclerosis (20, 21). As a membrane protein, ABCA1 was also reported to be degraded through the endosomal sorting complex required for transport/lysosome (ESCRT/lysosome) pathway (22). Ubiquitination is an important posttranslational modification of proteins and is widely involved in protein degradation, stability, translocation, and signaling pathway activation (23). However, the regulatory mechanisms underlying ABCA1 translocation and protein stability, especially associated with ubiquitination, have not yet been fully revealed.

Listerin E3 ubiquitin protein ligase 1 (Listerin) is an important member of the E3 ubiquitin ligase family with a typical Ring domain, and it’s homolog, LTN1, in yeast is reported to play an important role in regulating aberrant nascent polypeptides for proteasomal degradation (24). However, the role of Listerin in mammals and disease progression deserves further study. Macrophages internalize oxidized LDL (oxLDL) through scavenger receptors (e.g., CD36, SRA1). When their lipoprotein-engulfing capacity surpasses cholesterol efflux mechanisms — a hallmark of disrupted cholesterol homeostasis — these macrophages undergo phenotypic transition to foam cells, which subsequently drive the formation of the lipid-rich necrotic core characteristic of advanced atherosclerotic plaques. To investigate potential molecular regulators of this pathogenic cascade, we conducted transcriptomic profiling and identified *Listerin* as a markedly upregulated E3 ligase in cholesterol ester–stimulated macrophages. In a further examination of the role of Listerin in atherosclerosis, we conducted a series of molecular biology experiments and found that Listerin could bind to ABCA1 to catalyze its K63-linked ubiquitination at Lys1884 and Lys1957, which inhibited lysosomal degradation via the ESCRT/lysosome pathway. Our study identified the E3 ubiquitin ligase Listerin as a regulator of ABCA1 translocation from the cytomembrane and protein stability. We also found that this modification plays an important role in reducing lipid deposition in macrophages and the progression of atherosclerosis.

## Results

### Listerin expression increases in human and mouse atherosclerotic plaque tissues.

**Figure 1:**
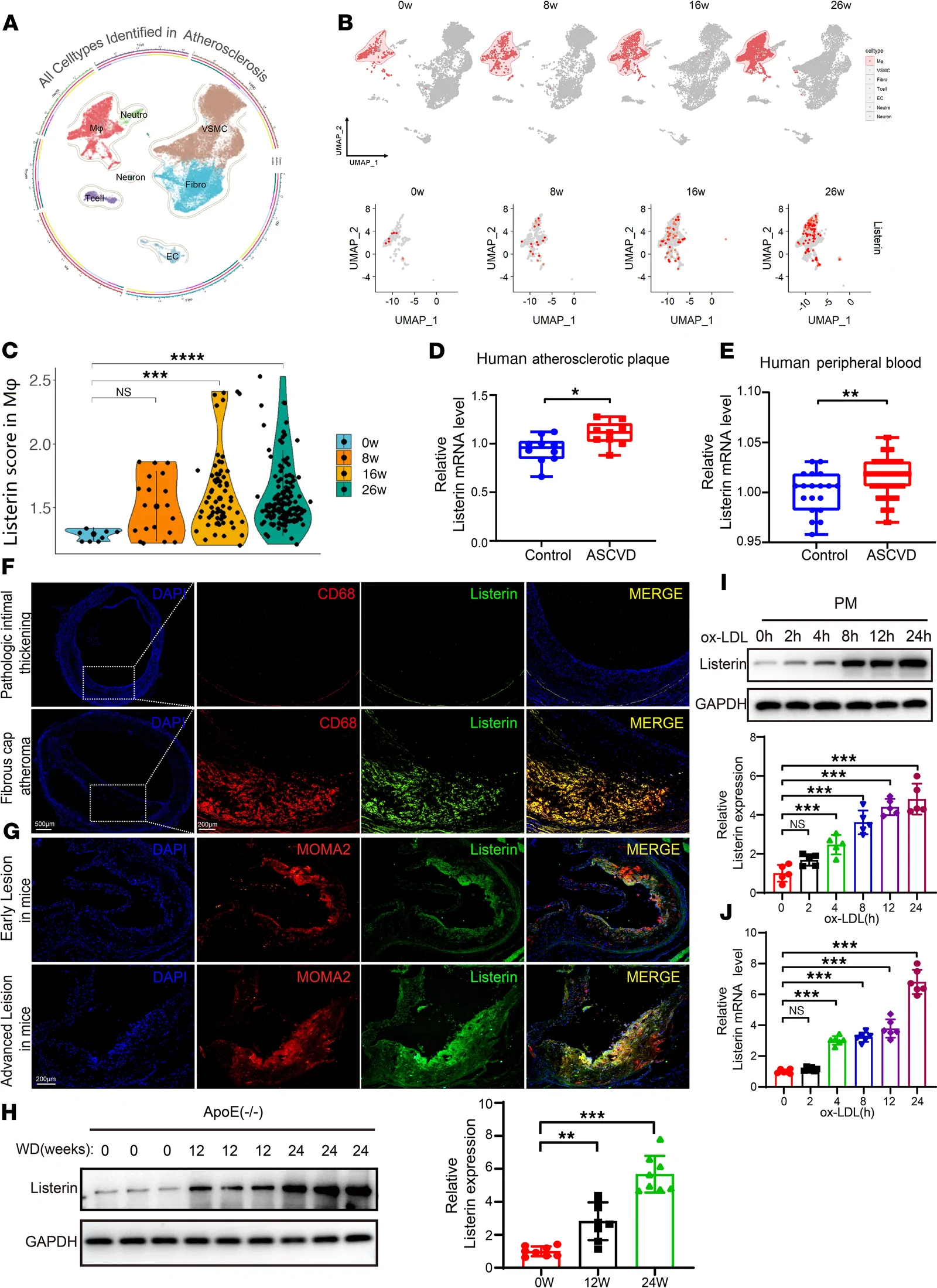
Listerin expression increases in human and mouse atherosclerotic plaque tissues. (**A**) All cell types identified in atherosclerotic plaque samples (*n* = 8) and 28,687 cells were obtained after filtering, with 7 of the cell types shown. (**B**) The proportion of macrophages was specifically increased with the progression of plaques. UMAP, uniform manifold approximation and projection. (**C**) The number and expression level of Listerin in macrophages (Mφ) from atherosclerotic plaques at different time points. (**D**) Data on *Listerin* mRNA expression in human atherosclerotic plaques were obtained from the GEO database (GSE57691). (**E**) Data on *Listerin* mRNA expression in human PBMCs were obtained from the GEO database (GSE23749). (**F**) Immunofluorescence staining for Listerin (green particles) and CD68 (red particles) in pathological intimal thickening and fibroatheroma in human coronary artery atherosclerotic plaques. *n* = 5 per group. Scale bars: 500 μm and 200 μm (enlarged insets). (**G**) Immunofluorescence staining for Listerin (green particles) and MOMA2 (red particles) in early lesions (WD for 8 weeks) and advanced lesions (WD for 24 weeks) of male mice. *n* = 8 per group. Scale bar: 200 μm. (**H**) Immunoblot analysis of Listerin expression in whole aorta lysates from *ApoE^–/–^* mice fed a WD for 0, 12, or 24 weeks. *n* = 8 per group. (**I**) Immunoblot analysis of Listerin expression in PMs after oxLDL (50 μg/mL) treatment. *n* = 5 per group. (**J**) Quantitative RT-PCR analysis of *Listerin* mRNA levels in PMs after oxLDL (50 μg/mL) treatment for the indicated durations. *n* = 6 per group. Data are presented as the mean ± SD. The Shapiro-Wilk method was used to test normal distributions, and statistical analysis was performed with an unpaired, 2-tailed Student’s *t* test (**D** and **E)**, Mann-Whitney *U* test (**C**), and 1-way ANOVA with Dunnett’s post hoc test (**H**, **I**, and **J**). Adjusted *P* values are provided for multiple-group comparisons. NS, *P* > 0.05; *P < 0.05, **P < 0.01, ***P < 0.001, and ***P < 0.0001. Each experiment was conducted at least 3 times independently.

Macrophage transition into foam cells under cholesterol ester accumulation drives atherosclerotic pathogenesis (25–27). To identify ubiquitination-related regulators of this process, we first performed RNA-Seq on oxLDL-treated Tsuchiya human monocytic leukemia line (THP-1)–derived macrophages alongside reanalysis of PBMC transcriptomes from patients with carotid atherosclerosis (Gene Expression Omnibus [GEO] GSE23746). Both datasets revealed *Listerin* upregulation in foam cells and patient monocytes (Supplemental Figure 1, A and B; supplemental material available online with this article; https://doi.org/10.1172/JCI186509DS1). To further explore the role of macrophage Listerin in atherosclerosis, we analyzed the time-series scRNA-Seq data from the Gene Expression Omnibus (GEO) database (GSE155513) and revealed cellular heterogeneity during the development of atherosclerotic plaque. There were 4 time points of plaque progression (Supplemental Figure 1C), including the 0 weeks group (0 wk), the 8 weeks group (8 wk), the 16 weeks group (16 wk), and the 26 weeks group (26 wk). Unbiased clustering of 28,687 cells from all samples revealed 18 clusters (Supplemental Figure 1C). Based on established lineage-specific marker genes (Supplemental Figure 1D), these clusters were assigned to 7 cell lineages, including endothelial cells (ECs) (*Pecam1, Cdh5*), vascular smooth muscle cells (VSMCs) (*Tagln, Acta2*), fibroblasts (*Serpinf1, Pdgfra*), and macrophages (*C1qa, Cd68*), among others (Figure 1A). The proportions of different cell types showed significant variations in the progression of atherosclerotic plaque (Supplemental Figure 1E). This includes transformations of SMCs and increased neutrophil infiltration, among other changes (Supplemental Figure 1F). Notably, macrophages obviously increased in both number and proportion with further aggravation of atherosclerotic plaques (Figure 1B and Supplemental Figure 1F). Intriguingly, *Listerin* expression within macrophages demonstrated progressive elevation across plaque development (Figure 1, B and C), a pattern concordant with the increased levels observed in human atherosclerotic plaques (GSE57691) and patient-derived PBMCs (GSE23746) (Figure 1, D and E). Therefore, the above results indicated that macrophage Listerin expression may be related to atherosclerosis progression.

To further investigate the association between macrophage expression of Listerin and atherosclerosis, we measured the expression of Listerin in coronary atherosclerotic plaques and found a strong positive correlation between macrophage Listerin and plaque progression (i.e., pathological intimal thickening [PIT], fibroatheroma) (Figure 1F and Supplemental Figure 1G), suggesting that Listerin plays an important role in the development of atherosclerosis. Parallel studies conducted in atherosclerotic mouse models (fed a Western diet [WD] for different durations) recapitulated this trend, with aortic Listerin expression significantly elevated in late-stage lesions characterized by dense macrophage infiltration (Figure 1G). This observation was further corroborated by immunoblot analysis of whole aortic lysates, which confirmed the upregulation of Listerin protein levels (Figure 1H). To further investigate the regulatory effect of oxLDL stimulation (a risk factor for atherosclerosis) on Listerin expression in macrophages, we isolated primary peritoneal macrophages (PMs). Following oxLDL stimulation, immunofluorescence assays demonstrated a time-dependent upregulation of Listerin expression in PMs (Supplemental Figure 1H). Subsequent immunoblotting (Figure 1I) and quantitative reverse transcription PCR (RT-PCR) analyses (Figure 1J) demonstrated significant increases in both protein and transcript levels of Listerin in oxLDL-treated PMs. Importantly, these findings were recapitulated in RAW264.7 macrophage cell lines, with parallel experiments showing consistent elevation of Listerin protein (Supplemental Figure 1I) and mRNA expression (Supplemental Figure 1J) under identical stimulation conditions. Taken together, these data suggest that Listerin is involved in the development of atherosclerosis and plays a potential role in this disease.

### Macrophage Listerin deficiency inhibits cholesterol efflux and aggravates foam cell formation.

**Figure 2:**
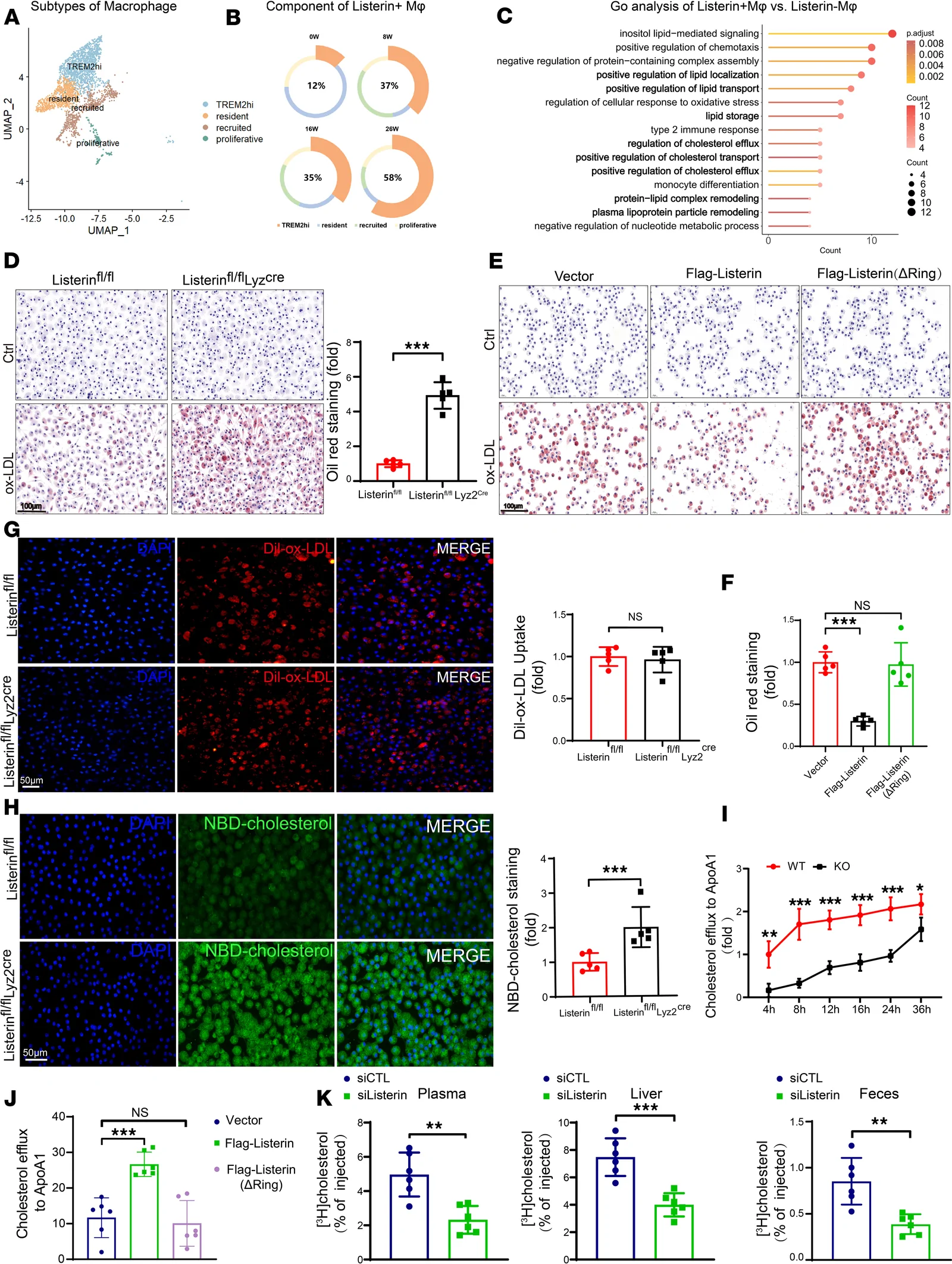
Macrophage Listerin KO inhibits cholesterol efflux and aggravates foam cell formation. (**A**) Macrophage subtypes were classified into 4 different subpopulations. (**B**) The percentage of Listerin^+^ macrophages among the 4 subtypes of macrophages as the atherosclerosis progressed. (**C**) GO pathway analysis of differentially expressed genes (DEGs) in Listerin^+^ macrophages. (**D**) Oil Red O–stained images and quantitation of PMs from *Listerin^fl/fl^* and *Listerin^fl/fl^ Lyz2^Cre^* mice after incubation with or without oxLDL (50 μg/mL) for 24 hours. *n* = 5 per group. Scale bar: 100 μm. Ctrl, control. (**E**) Oil Red O–stained images and (**F**) quantitation of PMs incubated with or without oxLDL (50 μg/mL) for 24 hours after adenovirus-mediated overexpression of Flag-Listerin and Flag–Listerin-ΔRing. *n* = 5 per group. Scale bar: 100 μm. (**G**) Immunofluorescence images and quantitation of PMs from *Listerin^fl/fl^* and *Listerin^fl/fl^ Lyz2^Cre^* mice treated with Dil-oxLDL (40 μg/mL) at 37°C for 4 hours. *n* = 5 per group. Scale bar: 50 μm. (**H**) Immunofluorescence images and quantitation of PMs preloaded with the NBD cholesterol and then incubated with ApoA1 for 4 hours. *n* = 5 per group. Scale bar: 50 μm. (**I**) Time course of ApoA1-mediated cholesterol efflux assay of PMs from *Listerin^fl/fl^* and *Listerin^fl/fl^ Lyz2^Cre^* mice. *n* = 3 per group. (**J**) ApoA1-mediated cholesterol efflux assay of PMs infected with the indicated adenovirus. *n* = 6 per group. (**K**) Percentage of [3^H^] cholesterol appearance in plasma, liver, and feces 48 hours after transplantation of cholesterol-loaded RAW264.7 macrophages transfected with either normal control or Listerin siRNA (*n* = 6 per group). Data are presented as the mean ± SD. The Shapiro-Wilk method was used to test normal distributions. Statistical analysis was performed using an unpaired, 2-tailed Student’s *t* test (**D**, **G**, and **K**), a Mann-Whitney *U* test (**H**), 2-way ANOVA followed by Šidák’s post hoc test (**I**), and 1-way ANOVA with Dunnett’s post hoc test (**F** and **J**). The adjusted *P* values are provided for multiple-group comparisons. NS, *P* > 0.05; *P < 0.05, **P < 0.01, and ***P < 0.001.

Macrophages exhibit functional plasticity during atherogenesis, dynamically engaging in lipid handling and inflammatory responses. To delineate Listerin’s spatial regulation, we performed a more detailed subtyping of macrophages (Figure 2A) and found that Listerin^+^ macrophages were primarily concentrated in the triggering receptor expressed on myeloid cells 2 (TREM2^hi^) macrophage subtype, and their proportion significantly increased with disease progression (Figure 2B). Gene ontology analysis further associated Listerin-expressing macrophages with lipid transport and cholesterol efflux signatures (Figure 2C), suggesting a potential role in cholesterol homeostasis. To functionally validate these observations, we generated macrophage-specific Listerin-KO mice (*Listerin^fl/fl^ Lyz2^Cre^*) via Cre-lox recombination to conditionally knock out exons 3–5 of the Listerin gene (Supplemental Figure 2A). Effective KO was confirmed by extracting PMs from *Listerin^fl/fl^* and *Listerin^fl/fl^ Lyz2^Cre^* mice (Supplemental Figure 2B).

On the basis of the aforementioned single-cell sequencing data, we next assessed the effects of Listerin on cholesterol metabolism and foam cell formation in detail. Oil Red O staining revealed exacerbated lipid accumulation in Listerin-deficient PMs under oxLDL stimulation (Figure 2D). Conversely, adenoviral overexpression of WT Listerin — but not its E3 ligase–deficient mutant (ΔRing) — attenuated foam cell formation in PMs (Figure 2, E and F, and Supplemental Figure 2C). Furthermore, we restored Listerin protein expression in Listerin-deficient PMs through adenovirus infection. Reconstitution of Listerin restored lipid deposition upon stimulation with oxLDL, whereas the truncated mutant Listerin-ΔRing did not (Supplemental Figure 2D). These findings position Listerin as a ubiquitination-dependent regulator of macrophage lipid metabolism.

Given the established paradigm that foam cell formation stems from disrupted cholesterol efflux–lipoprotein uptake equilibrium (28–30), we stimulated PMs with fluorescence-labeled, oxidized low-density lipoprotein (Dil-oxLDL) to systematically dissect Listerin’s mechanistic contribution. We found that Listerin deficiency did not impair modified lipoprotein uptake capacity in PMs (Figure 2G). Concurrently, de novo lipogenesis assays revealed no significant alteration in lipid synthesis pathways in Listerin-deficient macrophages (Supplemental Figure 2E). These combined observations prompted us to hypothesize that Listerin might regulate cholesterol efflux mechanisms rather than influence lipoprotein uptake or lipid biosynthesis processes. Cholesterol efflux from plaque macrophages is also known as RCT, which plays a key role in foam cell formation (12, 31–34) by reducing lipid deposition of macrophages in atherosclerotic lesions. Thus, we first assessed the effects of Listerin on cholesterol efflux in vitro and found that the Listerin deficiency resulted in accumulated nitrobenzoxadiazole (NBD) cholesterol in the PMs was significantly increased (Figure 2H), and a time-dependent reduction of cholesterol efflux to ApoA1 (Supplemental Figure 2F and Figure 2I). Accordingly, cholesterol efflux was detected in Listerin-overexpressing PMs. Listerin overexpression promoted cholesterol efflux to ApoA1, but not its E3 ligase–deficient mutant (ΔRing) (Figure 2J). Furthermore, we found that reconstitution of Listerin in Listerin-deficient PMs restored cholesterol efflux in a Ring domain–dependent manner (Supplemental Figure 2G), mirroring the foam cell formation phenotypes. Next, we studied the effect of Listerin deficiency on RCT in vivo. RAW264.7 macrophages transfected with control or Listerin siRNA were loaded with oxLDL and transplanted into the peritoneal cavity of mice. The amount of labeled cholesterol exported from macrophages with Listerin knockdown to the plasma, liver, and feces was reduced compared with that of control macrophages (Figure 2K). Altogether, these findings suggest that Listerin can reduce lipid accumulation by promoting cholesterol efflux, thereby ameliorating macrophage-derived foam cell formation.

### Macrophage Listerin deficiency promotes lipid accumulation and foam cell formation through downregulation of ABCA1 expression.

**Figure 3:**
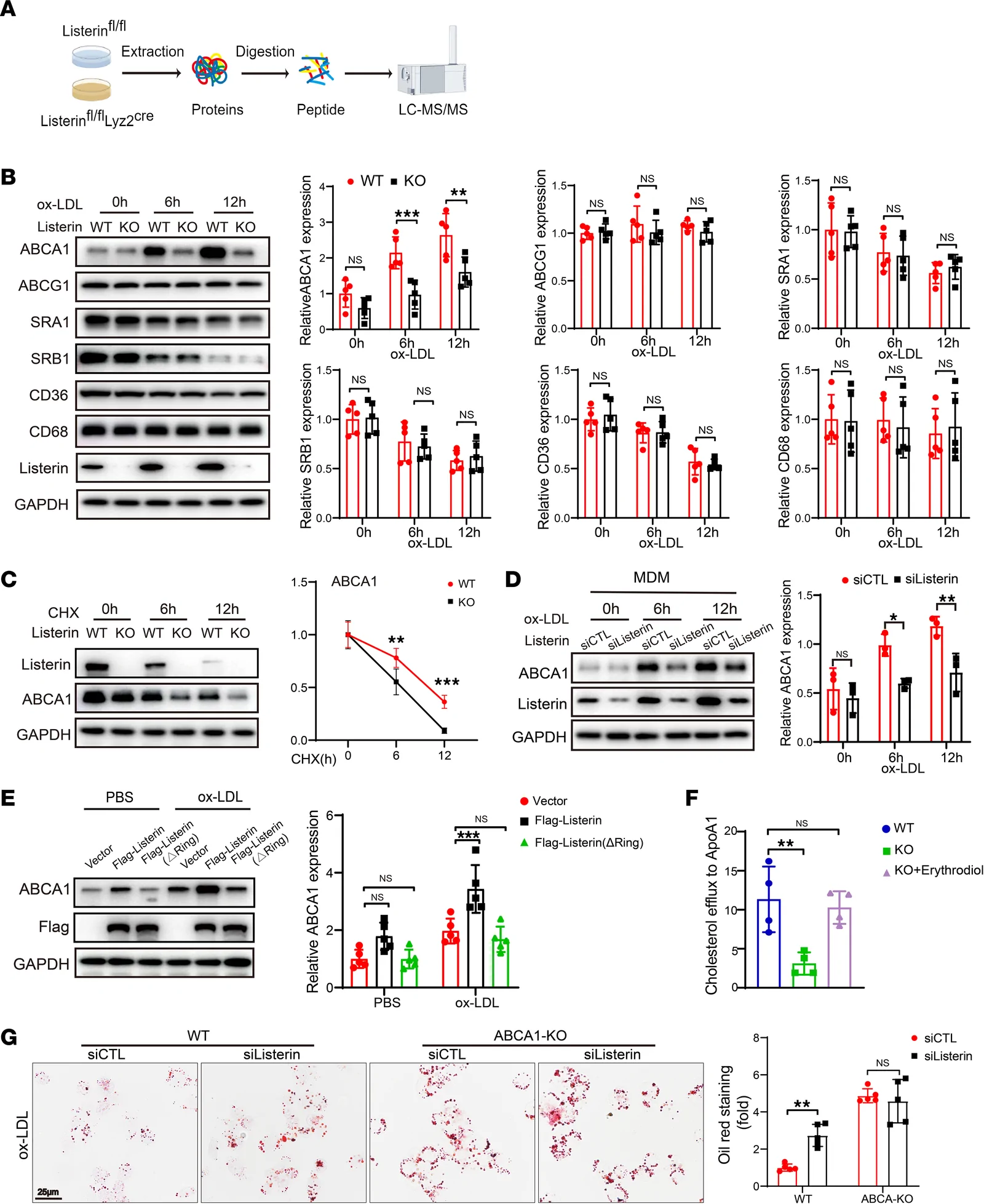
Listerin KO promotes lipid accumulation and foam cell formation through downregulation of ABCA1. (**A**) Diagram of 4D-FastDIA–based quantitative proteomics of PMs isolated from *Listerin^fl/fl^* and *Listerin^fl/fl^ Lyz2^Cre^* mice. (**B**) Immunoblot analysis of the indicated proteins in *Listerin^fl/fl^* and *Listerin^fl/fl^ Lyz2^Cre^* PMs after oxLDL treatment. *n* = 5 per group. (**C**) Immunoblot and quantitative analyses of ABCA1 expression in PMs obtained from *Listerin^fl/fl^* and *Listerin^fl/fl^ Lyz2^Cre^* mice. The cells were treated with oxLDL for 12 hours and then treated with cycloheximide (CHX) (50 μg/mL) for various durations. (**D**) Immunoblot analysis of ABCA1 expression in MDMs (derived from PBMCs induced by M-CSF [50 ng/mL] for 7 days) after Listerin knockdown. *n* = 3 per group. (**E**) Immunoblot analysis of ABCA1 expression after adenovirus-mediated overexpression of Flag-Listerin or Flag-Listerin (ΔRing) in PMs. *n* = 5 per group. (**F**) ApoA1-mediated cholesterol efflux assay of PMs isolated from *Listerin^fl/fl^* and *Listerin^fl/fl^ Lyz2^Cre^* mice. PMs were preincubated with or without the ABCA1 agonist erythrodiol. *n* = 4 per group. (**G**) Oil Red O–stained images and quantitation of WT and ABCA1-KO THP-1 cells transfected with siCTL or siListerin. *n* = 5 per group. Scale bar: 25 μm. Data are presented as the mean ± SD. The Shapiro-Wilk method was used to test normal distributions. Significance was determined by 2-way ANOVA followed by Tukey’s post hoc test (**F**), 2-way ANOVA followed by Šidák’s post hoc test (**B**–**D** and **G**), and 1-way ANOVA with Dunnett’s post hoc test (**F**). The adjusted *P* values are provided for multiple-group comparisons. NS, *P* > 0.05; *P < 0.05, **P < 0.01, and ***P < 0.001.

To delineate the molecular mechanism underlying Listerin-mediated cholesterol efflux, we performed quantitative proteomics analysis in macrophages isolated from Listerin-deficient mice and control mice (Figure 3A). This analysis revealed that ABCA1 — a master regulator of cholesterol transport — was significantly downregulated upon Listerin ablation (Table 1). To verify the proteomics results, we examined some lipid endocytosis–associated receptors such as the class A1 scavenger receptor (SRA1), CD36, and CD68 (35). Cholesterol efflux receptors, ABCA1, ATP-binding cassette transporter G1 (ABCG1), and scavenger receptor type B class I (SRB1) were also analyzed (31). Interestingly, following oxLDL stimulation, Listerin deficiency significantly reduced ABCA1 protein expression in PMs, while other receptors remained unaffected (Table 1). Notably, *ABCA1* mRNA levels showed no alterations (Supplemental Figure 3A), suggesting that Listerin regulated ABCA1 at the posttranscriptional level. Additionally, cycloheximide chase assays demonstrated accelerated ABCA1 degradation in KO macrophages, establishing Listerin’s role in posttranslational stabilization (Figure 3C). Consistently, we next isolated PBMCs from both control individuals and patients with ASCVD, and differentiated them into peripheral blood monocyte–derived macrophages (MDMs) using macrophage CSF (M-CSF). RNA interference–mediated knockdown of Listerin in these cells demonstrated significant downregulation of ABCA1 expression (Figure 3D). This finding was recapitulated in bone marrow–derived macrophages (BMDMs) from Listerin-KO mice, which showed a concordant reduction in ABCA1 protein levels (Supplemental Figure 3B). On the contrary, adenovirus-mediated overexpression of Listerin, but not Listerin-ΔRing, increased ABCA1 protein expression in PMs (Figure 3E). Furthermore, reconstitution of Listerin in Listerin-KO PMs restored oxLDL-induced ABCA1 expression, whereas the catalytically inactive Listerin-ΔRing–mutant failed to rescue this phenotype (Supplemental Figure 3C), establishing a strict structure-function relationship between Listerin’s E3 ligase activity and ABCA1 stabilization.

To establish causality between Listerin/ABCA1 axis dysfunction and foam cell formation, we applied pharmacological and genetic approaches. Erythrodiol is a selective inhibitor of ABCA1 protein degradation, and our results confirmed that erythrodiol could upregulate the expression of ABCA1 protein without affecting the expression of ABCG1 or SRB1 (Supplemental Figure 3D). Next, we assessed cholesterol efflux and foam cell formation in PMs isolated from *Listerin^fl/fl^* and *Listerin^fl/fl^ Lyz2^Cre^* mice, and the results indicated that Listerin-deficient PMs had significantly increased foam cell formation and inhibited cholesterol efflux, which was abrogated specifically by pretreatment using the ABCA1 agonist erythrodiol (Figure 3F and Supplemental Figure 3, E and F). Crucially, we constructed an ABCA1-KO THP-1 cell using the CRISPR/Cas9 system and found that Listerin deficiency did not further increase lipid deposition in ABCA1-KO cells (Figure 3G). These findings collectively position Listerin as a ubiquitin-dependent stabilizer of ABCA1, orchestrating macrophage cholesterol efflux to mitigate foam cell pathogenesis.

### Listerin inhibits the degradation of ABCA1 through the ESCRT/lysosome pathway.

ABCA1 protein degradation has a precise regulatory mechanism, and excessive or misfolded ABCA1 may be degraded. Notably, ABCA1 protein degradation includes 2 important pathways: the calpain-mediated degradation pathway (36, 37) and the ubiquitin-mediated degradation pathway (22). Ubiquitin-mediated degradation of ABCA1 can be divided into lysosome and non-lysosome pathways (38). Ubiquitin is mostly considered a sorting protein for proteasome degradation. A growing body of evidence shows that this molecule also labels membrane proteins for lysosomal degradation, in which the ESCRT pathway serves as a dominant mechanism (22, 39, 40). Disruption of ESCRT significantly delays the degradation of cell-surface-resident ABCA1 (22).

**Figure 4:**
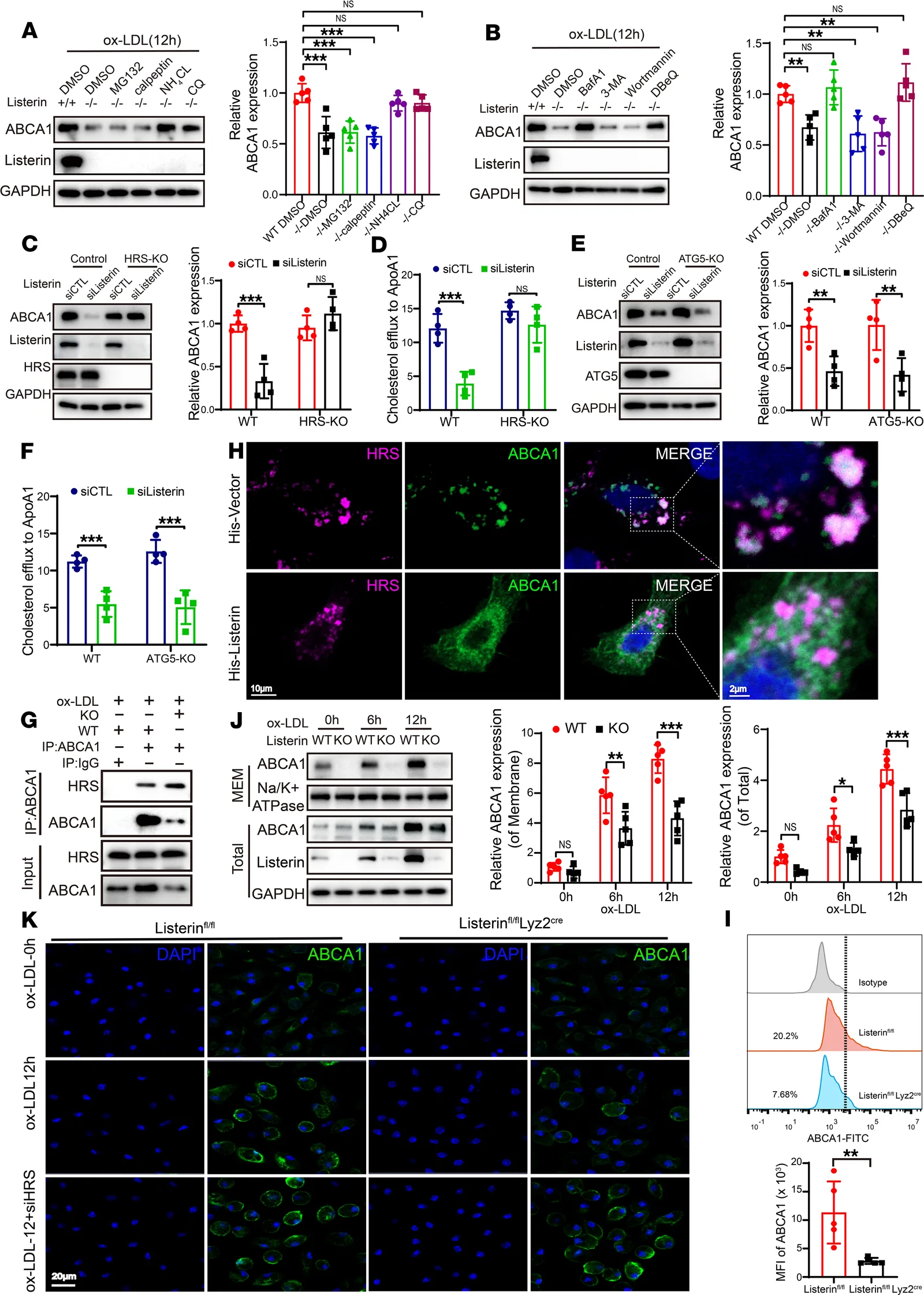
Listerin inhibits the degradation of ABCA1 through the ESCRT lysosome pathway. (**A** and **B**) Immunoblot analysis of ABCA1 expression in PMs from *Listerin^fl/fl^* and *Listerin^fl/fl^ Lyz2^Cre^* mice treated for 12 hours with oxLDL (50 μg/mL) and proteasome/lysosome inhibitors (MG132, calpeptin, chloroquine, NH_4_Cl, bafilomycin A1, 3-MA, wortmannin, DBeQ). *n* = 5 per group. (**C**) ABCA1 immunoblot in control/HRS-KO THP-1 cells with siCTL/Listerin silencing and oxLDL stimulation. *n* = 4 per group. (**D**) ApoA1-mediated cholesterol efflux in HRS-KO THP-1 cells transfected with siCTL/siListerin. *n* = 4 per group. (**E**) Immunoblotting for ABCA1 was performed in control/ATG5-KO THP-1 cells with siCTL/Listerin silencing. *n* = 4 per group. (**F**) Cholesterol efflux in ATG5-KO THP-1 cells transfected with siCTL/siListerin. *n* = 4 per group. (**G**) Co-IP of ABCA1-HRS interaction in oxLDL-treated PMs. An equal amount of nonspecific antibody was used as a negative control. (**H**) Confocal imaging of Flag-HRS/GFP-ABCA1 colocalization with/without His-Listerin overexpression. in HeLa cells. Scale bars: 10 μm and 2 μm (enlarged insets). (**I**) Flow cytometric analysis of membrane ABCA1 in PMs from *Listerin^fl/fl^* and *Listerin^fl/fl^ Lyz2^Cre^* mice. *n* = 5 per group. (**J**) Immunoblot analysis of membrane (MEM) and total ABCA1 in PMs from *Listerin^fl/fl^* and *Listerin^fl/fl^ Lyz2^Cre^* mice. *n* = 5 per group. (**K**) Confocal microscopic images of ABCA1 expression in PMs from *Listerin^fl/fl^* and *Listerin^fl/fl^ Lyz2^Cre^* mice. HRS was silenced in PMs, which were then stimulated with oxLDL (50 μg/mL). *n* = 5 per group. Scale bar: 20 μm. Data are presented as the mean ± SD. The Shapiro-Wilk method was used to test normal distributions. Data analysis was performed using 1-way ANOVA followed by Dunnett’s post hoc test (**A** and **B**), unpaired, 2-tailed Student’s *t* test (**I**), and 2-way ANOVA followed by Šidák’s post hoc test for the other panels. The adjusted *P* values are provided for multiple-group comparisons. NS, *P* > 0.05; *P < 0.05, **P < 0.01, and ***P < 0.001. Each experiment was repeated at least 3 times independently.

To define Listerin’s role in ABCA1 regulation, we carried out a series of experiments, and the results showed that the protein degradation of ABCA1 in Listerin-deficient PMs was restored by the lysosomal inhibitors chloroquine, NH_4_Cl, and bafilomycin A1 (BafA1), but not by the proteasome inhibitor MG132 or the calpain inhibitor calpeptin (Figure 4, A and B). In conclusion, the loss of Listerin in macrophages mediated ABCA1 degradation through lysosomes. We investigated the lysosome sorting mechanism in Listerin-deficient PMs using the autophagy inhibitor 3MA and wortmannin, or the ESCRT inhibitor DBEQ (blocks the formation of multivesicular bodies). Unexpectedly, the degraded ABCA1 was restored by DBeQ (Figure 4B). We also designed a specific siRNA for hepatocyte growth factor–regulated tyrosine kinase substrate (HRS), a subunit of ESCRT-0 that plays an important role in the ESCRT system (39, 41), and this siRNA resulted in significantly lower expression of endogenous HRS (Supplemental Figure 4A). The results showed that the degradation of ABCA1 protein by Listerin-deficient PMs was restored after HRS-specific siRNA application (Supplemental Figure 4B). Collectively, these findings provide initial evidence that Listerin deficiency accelerated ABCA1 degradation via the ESCRT-dependent lysosomal sorting machinery.

To further verify the correlation between the ESCRT system and ABCA1 degradation, we used the CRISPR/Cas9 system to knock out HRS in THP-1 cells. Consistently, the decrease in ABCA1 protein expression and cholesterol efflux resulting from Listerin deficiency were restored after HRS deletion (Figure 4, C and D). In contrast, ATG5-KO (ATG5 is an essential autophagy-related protein that regulates autophagy formation; ref. 42) THP-1 cells still showed decreased ABCA1 expression and cholesterol efflux after Listerin deficiency (Figure 4, E and F). We observed similar results in HRS-KO or ATG5-KO HEK293T cells (Supplemental Figure 4, C and D). Mechanistic interrogation revealed that Listerin deficiency increased ABCA1-HRS binding despite reducing total ABCA1 levels (Figure 4G), whereas Listerin overexpression diminished this interaction in both co-IP (Supplemental Figure 4E) and immunofluorescence assays (Figure 4H). Parallel lysosomal tracking demonstrated enhanced ABCA1 accumulation in lysosomes upon Listerin KO (Supplemental Figure 4F). These complementary approaches demonstrate that Listerin stabilized ABCA1 by blocking its recognition by the ESCRT complex, thereby preventing lysosome-mediated degradation.

Beyond stabilizing ABCA1 protein levels, Listerin was found to regulate ABCA1 membrane dynamics, and thus cell-surface-resident ABCA1 was investigated. We used flow cytometry to examine the changes in ABCA1 expression on the cell membrane after Listerin KO. The results showed that the fluorescence intensity of membrane ABCA1 was significantly reduced in Listerin-deficient macrophages (Figure 4I), whereas overexpression of Listerin significantly enhanced the fluorescence intensity of membrane ABCA1 (Supplemental Figure 4, G and H). Complementarily, we extracted cell-surface-resident proteins from PMs of *Listerin^fl/fl^* and *Listerin^fl/fl^ Lyz2^Cre^* mice. Western blotting (Figure 4J) and immunofluorescence (Figure 4K) analyses demonstrated preferential degradation of membrane-resident ABCA1 over total cellular pools in KO cells. Notably, HRS knockdown reversed this surface depletion, as evidenced by restored membrane ABCA1 expression in both immunofluorescence (Figure 4K) and immunoblot (Supplemental Figure 4I) analyses. These findings collectively demonstrate Listerin’s role in preserving ABCA1 membrane residency by blocking ESCRT-mediated lysosomal sorting.

Although yeast LTN1 (Listerin homolog) participates in ribosome-associated quality control (RQC) through proteasomal targeting of aberrant polypeptides (43–45), genetic disruption of RQC via nuclear export mediator factor (NEMF) knockdown — the central player of the RQC system (45, 46) — failed to rescue ABCA1 degradation in Listerin-deficient macrophages (Supplemental Figure 4, J and K). CRISPR-engineered NEMF-KO THP-1 cells similarly maintained ABCA1 loss and cholesterol efflux defects upon Listerin deletion (Supplemental Figure 4, L and M), confirming mechanistic independence from RQC pathways. This multitiered analysis establishes Listerin as a specialized regulator of ABCA1 trafficking, operating through ESCRT lysosomal inhibition rather than canonical quality control mechanisms.

### Listerin targets ABCA1.

**Figure 5:**
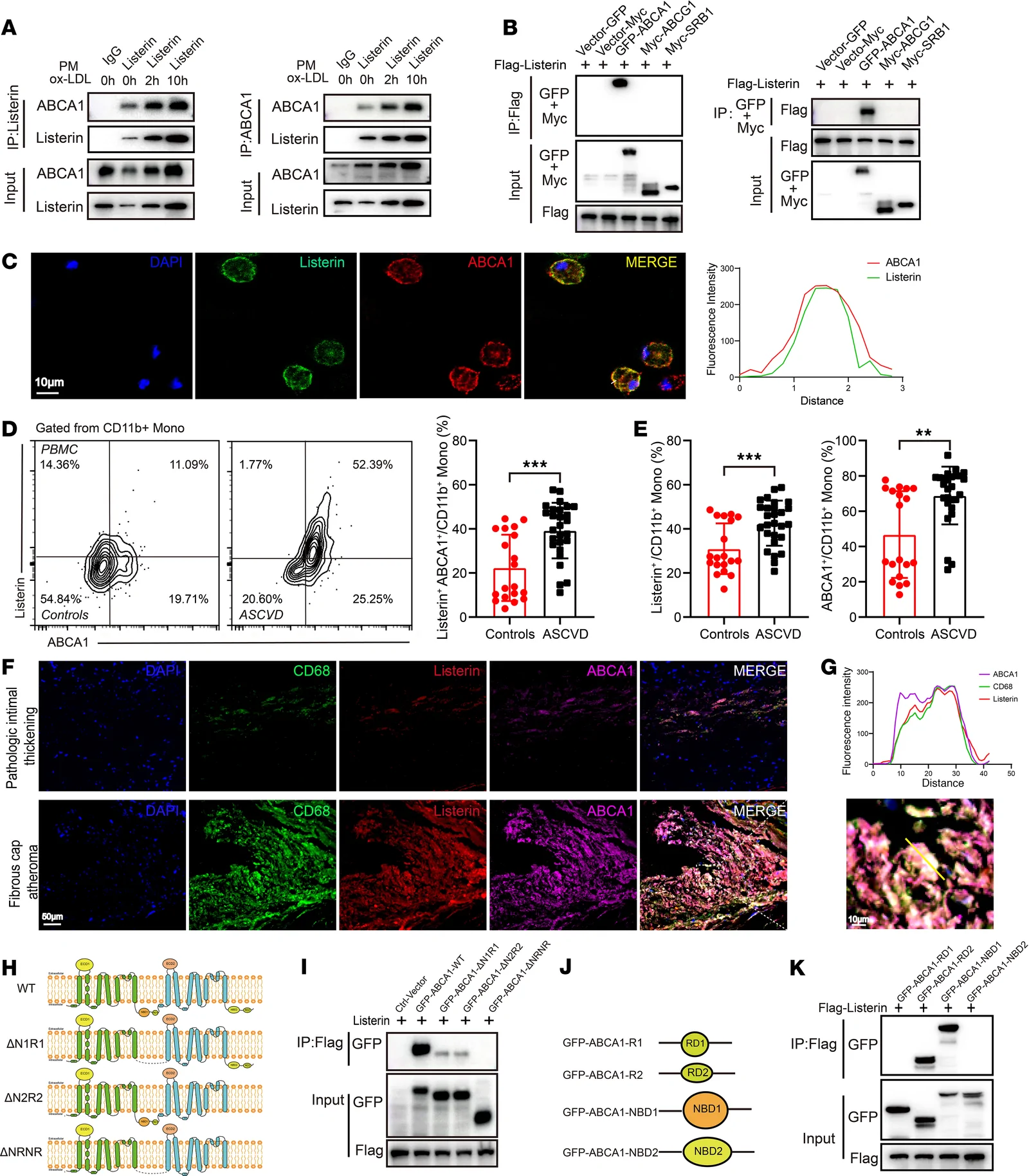
Listerin targets ABCA1. (**A**) Co-IP assay of endogenous shows that Listerin interacted with ABCA1 in PMs treated with oxLDL (50 μg/mL) for the indicated durations. An equal amount of nonspecific antibody was used as a negative control. (**B**) Co-IP assay of Flag-Listerin with GFP-ABCA1, Myc-ABCG1, or Myc-SRB1 in HEK293T cells. (**C**) Confocal microscopic images and fluorescence intensity analysis for Listerin and ABCA1 in primary PMs after oxLDL incubation (50 μg/mL). Scale bar: 10 μm. (**D**) Expression of Listerin and ABCA1 in CD11b^+^ monocytes from PBMCs from healthy individuals (*n* = 19) and patients with ASCVD (*n* = 27). The graph shows the proportion of Listerin^+^ABCA1^+^CD11b^+^ monocytes among total CD11b^+^ monocytes. (**E**) Expression of Listerin and ABCA1 was measured in CD11b^+^ monocyte from PBMCs from healthy individuals (*n* = 19) and patients with AS (*n* = 27). (**F**) Immunofluorescence staining for Listerin (red particles), ABCA1 (pink particles), and CD68 (green particles) in pathological intimal thickening and fibroatheroma in human coronary artery atherosclerotic plaques. Scale bars: 50 μm and 10 μm (enlarged inset). (**G**) Fluorescence intensity analysis for Listerin (red particles), ABCA1(pink particles), and CD68 (green particles) in fibroatheroma in human coronary artery atherosclerotic plaques. (**H** and **J**) Topological diagrams of human ABCA1 and mutants. (**I** and **K**) Co-IP assay of the interaction of Flag-Listerin with GFP-ABCA1 (WT) and the ABCA1 truncation mutants in HEK293T cells. Data are presented as the mean ± SD. The Shapiro-Wilk method was used to test normal distributions. Data analysis was performed using the Mann-Whitney *U* test (**D** and **E)**. The adjusted *P* values are provided for multiple-group comparisons. NS, *P* > 0.05; **P < 0.01 and ***P < 0.001. Each experiment was repeated at least 3 times independently.

To elucidate the structural basis of Listerin-ABCA1 interaction, we performed systematic domain-mapping experiments. Time-gradient co-IP assays in oxLDL-stimulated PMs revealed progressive enhancement of endogenous Listerin-ABCA1 binding (Figure 5A), consistent with prior observations of cholesterol-dependent transporter regulation. Specificity analysis in HEK293T cells demonstrated that Listerin exclusively coprecipitated with ABCA1, but not with ABCG1 or SRB1 (Figure 5B and Supplemental Figure 5A), whereas confocal microscopy confirmed their membrane colocalization under atherogenic conditions (Figure 5C). To further validate the clinical association between Listerin and ABCA1 in human macrophages, we performed flow cytometric analysis of PBMCs isolated from control individuals and patients with ASCVD and found a marked upregulation of both Listerin and ABCA1 expression in CD11b^+^ monocyte subsets (Figure 5, D and E). We further validated this expression profile in PBMCs from control individuals and patients with ASCVD and found that ASCVD-derived PBMCs exhibited higher Listerin and ABCA1 protein levels than did control PBMCs (Supplemental Figure 5B). Complementing these cellular findings, multiparametric immunofluorescence analysis of human coronary artery plaques demonstrated coordinated upregulation of both Listerin and ABCA1 proteins in plaque-associated macrophages during atherosclerotic progression (Figure 5, F and G). These findings suggest a robust correlation between Listerin and ABCA1 in macrophages, indicating that Listerin likely modulates foam cell formation through its regulatory effects on ABCA1. Then, dose-dependent overexpression of Listerin in HEK293T cells confirmed Listerin-mediated ABCA1 stabilization (Supplemental Figure 5C), which was abrogated by the catalytic mutants Listerin-ΔRing and Listerin-C/A (Supplemental Figure 5D), implicating ubiquitination-dependent regulation.

To explore the binding domains of ABCA1 that are necessary for its interaction with Listerin, several GFP-ABCA1 truncated mutants were constructed, including the deletion mutants GFP-ABCA1-ΔN1R1 (in which the NBD1 and RD1 domains were deleted), GFP-ABCA1-ΔN2R2 (in which the NBD2 and RD2 domains were deleted), and GFP-ABCA1-ΔNRNR (in which the full NBD/RD domains were deleted) (Figure 5H), and some other truncated plasmids, such as GFP-ABCA1-RD1, RD2, NBD1, and NBD2 (Figure 5J). Co-IP and immunofluorescence experiments show that the binding of ΔN1R1 and ΔN2R2 mutants to Listerin was markedly reduced, while the ΔNRNR mutant almost completely lost Listerin interaction (Figure 5I and Supplemental Figure 5E). Importantly, both GFP-ABCA1-RD2 (containing only the RD2 domain) and GFP-ABCA1-NBD1 (containing only the NBD1 domain) maintained effective coprecipitation with Flag-Listerin (Figure 5K). On the basis of these results, we conclude that Listerin interacted with the RD2 and NBD1 domains of ABCA1, modulating its stability through spatially defined ubiquitination to regulate cholesterol efflux.

### Listerin catalyzes K63-linked polyubiquitination of ABCA1 at the residues Lys1884 and Lys1957 to inhibit foam cell formation.

**Figure 6:**
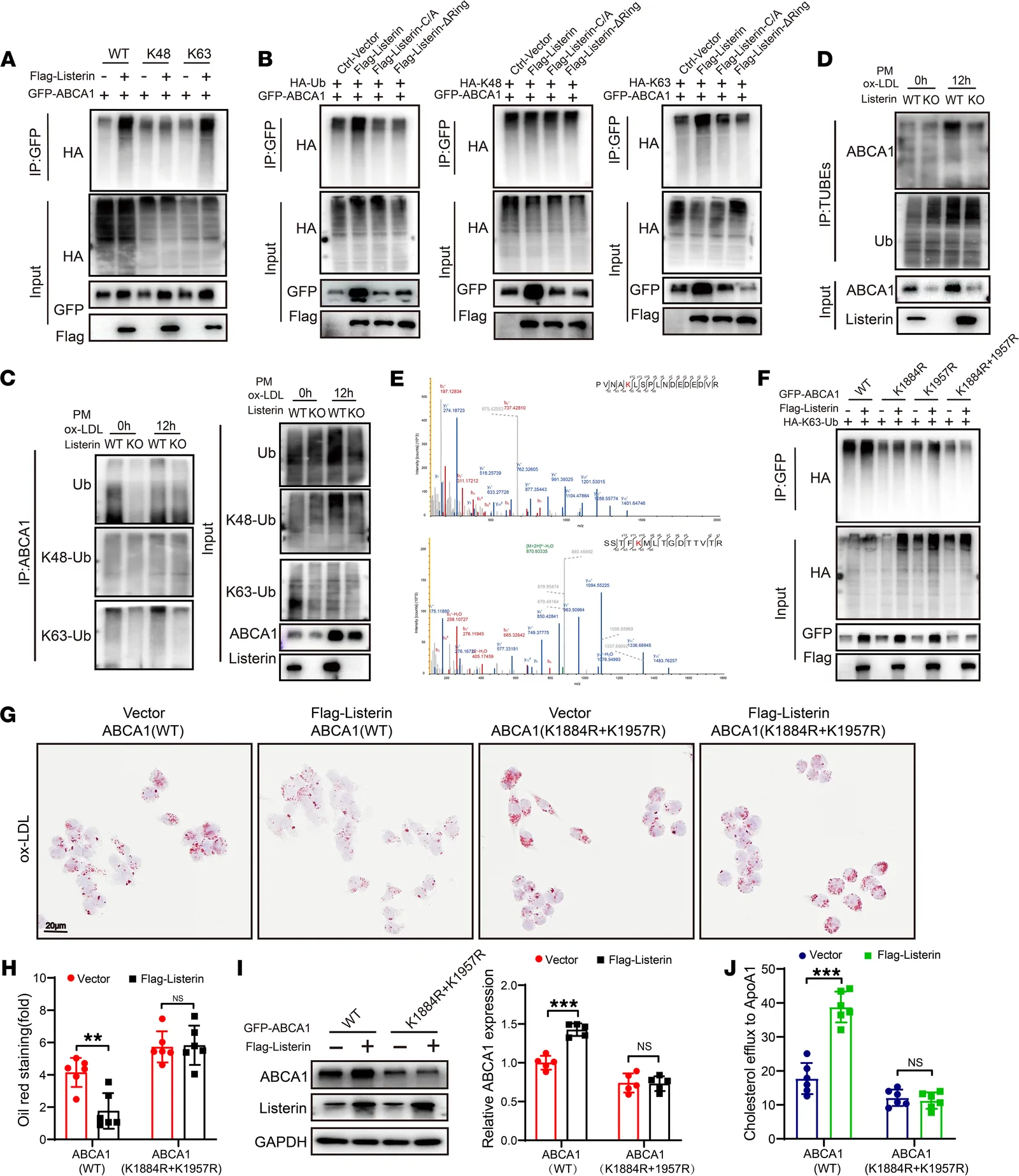
Listerin catalyzes K63-linked polyubiquitination of ABCA1 at residues Lys1884 and Lys1957 to inhibit foam cell formation. (**A**) Co-IP assay of ABCA1 polyubiquitination in HEK293T cells transfected with GFP-ABCA1, Flag-Listerin, HA-ubiquitin (WT), or HA-ubiquitin (K48 or k63). (**B**) Co-IP assay of ABCA1 polyubiquitination in HEK293T cells transfected with GFP-ABCA1, HA-ubiquitin (WT), HA-ubiquitin (K48 or K63), as well as a control vector, Flag-Listerin (WT), Flag-Listerin (C/A), or Flag-Listerin-ΔRing. (**C**) Co-IP assay of endogenous ABCA1 polyubiquitination in PMs from *Listerin^fl/fl^* *and Listerin^fl/fl^ Lyz2^Cre^* mice after being stimulated with oxLDL for 12 hours. (**D**) Co-IP assay of ABCA1 polyubiquitination after ubiquitin (TUBE) pull-downs in PMs. (**E**) LC-MS spectra analysis identified the ubiquitin modification of ABCA1 at lysine residues K1884 and K1957. (**F**) Co-IP analysis of the polyubiquitination of ABCA1 (WT) and its mutants in HEK293T cells transfected with GFP-ABCA1 (WT or mutants), Flag-Listerin, or HA-ubiquitin (K63). (**G**) Oil Red O–stained images and (**H**) quantitation analysis of RAW264.7 macrophages transfected with Flag-Listerin and GFP-ABCA1 (WT) or GFP-ABCA (K1884R and K1957R), and then incubated with oxLDL (50 μg/mL) for 24 hours. *n* = 6 per group. Scale bar: 20 μm. (**I**) Immunoblot analysis of GFP-ABCA1 and GFP-ABCA1(K1884 and K1957) expression in RAW246.7 macrophages. *n* = 5 per group. (**J**) ApoA1-mediated cholesterol efflux assay of RAW246.7 macrophages transfected with Flag-Listerin, GFP-ABCA1, or GFP-ABCA1(K1884 and K1957). *n* = 6 per group. Data are presented as the mean ± SD. The Shapiro-Wilk method was used to test normal distributions. Data analysis was performed with 2-way ANOVA followed by Šidák’s post hoc test. The adjusted *P* values are provided for multiple-group comparisons. NS, *P* > 0.05; **P < 0.01 and ***P < 0.001. Each experiment was repeated at least 3 times independently.

Ubiquitination is a crucial posttranslational modification of proteins that are involved in protein degradation, stability, translocation, and signaling pathway activation (23). ABCA1 can be ubiquitinated, followed by proteasomal and lysosomal degradation (37). Building on the established interaction between Listerin and ABCA1, we investigated its enzymatic role in ABCA1 ubiquitination. Coexpression of Listerin with ABCA1 and ubiquitin variants in HEK293T cells revealed preferential induction of K63-linked (versus K48-linked) polyubiquitination, dependent on intact E3 ligase activity (Figure 6, A and B). This pattern was recapitulated endogenously, with Listerin-KO macrophages showing reduced ABCA1 K63 ubiquitination without affecting K48-linked modifications (Figure 6C). Furthermore, we performed tandem ubiquitin-binding entity (TUBE) pull-down assays to purify the ubiquitinated substrates. Western blotting analyses showed that the polyubiquitination of ABCA1 was reduced in Listerin-deficient macrophages (Figure 6D).

To identify the lysine residues of ABCA1 responsible for Listerin-mediated polyubiquitination, we performed liquid chromatography–mass spectrometry (LC-MS) analysis of HEK293T cells, in which GFP-ABCA1 was cotransfected with Flag-Listerin and HA-ubiquitin, or GFP-ABCA1 only with HA ubiquitin. Comparative analysis revealed 2 intracellular lysine residues (K1884 and K1957) that had high-confidence scores and changed markedly after overexpression of Listerin compared with basal conditions (Figure 6E). Next, we performed precise structural localization of these ubiquitination sites by integrating the ABCA1 cryo-EM structure (PDB:7TBY) with AlphaFold2 predictions (Supplemental Figure 5F). Based on these results, we constructed the ABCA1 mutants K1884R and K1957R, in which the lysine residues were replaced with arginine. At the same time, we also constructed the ABCA1 mutants K1314R, K1189R, and K2023R according to the LC-MS analysis to verify whether other lysine sites participated in Listerin-mediated ABCA1 ubiquitination. Subsequently, the mutants were transfected into HEK293T cells, and analysis revealed that Listerin-mediated K63-linked polyubiquitination of ABCA1 increased markedly in cells containing the K1314R, K1189R, and K2023R mutants (Supplemental Figure 5G). However, in the K1884R and K1957R mutants, Listerin-mediated polyubiquitination of K63-linked ABCA1 increased slightly. Notably, mutations at both loci (K1884R and K1957R) restored K63-linked ubiquitination of ABCA1 mediated by Listerin (Figure 6F). Functional studies in RAW264.7 macrophages demonstrated loss of Listerin’s lipid-reducing effects when expressing these ABCA1 mutants, as evidenced by unabated Oil Red O staining (Figure 6, G and H), unchanged ABCA1 protein levels (Figure 6I), and impaired cholesterol efflux rescue (Figure 6J). Correspondingly, in the in vivo RCT experiments, because of the K1884 and K1957 mutations in ABCA1, Listerin was unable to continue promoting the ubiquitination and stabilization of ABCA1. As a result, the cholesterol efflux capacity of ABCA1-K1884/K1957 was weakened compared with that of WT ABCA1 (Supplemental Figure 5H). Collectively, these findings delineate a nonproteolytic ubiquitination mechanism whereby Listerin catalyzes K63-linked polyubiquitination of ABCA1 at residues Lys1884 (located in the flexible cytoplasmic loop adjacent to the NBD2 domain) and Lys1957 (located in the solvent-exposed region of the cytosolic NBD2 domain) to enhance cholesterol efflux capacity and mitigate foam cell pathogenesis.

### Listerin deficiency aggravates the development of atherosclerosis in vivo.

**Figure 7:**
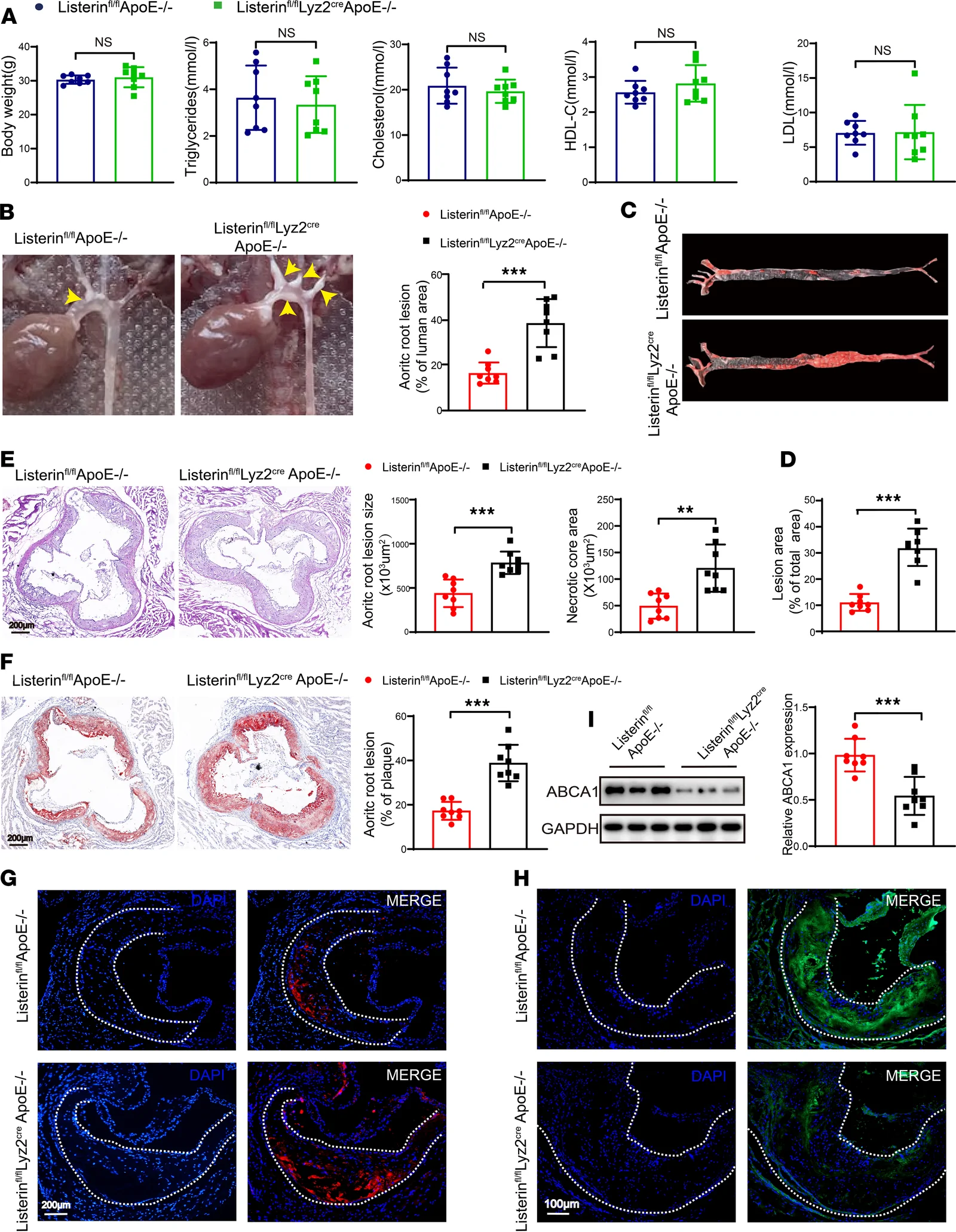
Listerin KO aggravates the development of atherosclerosis in vivo. Male *ApoE^–/–^ Listerin^fl/fl^* and *ApoE^–/–^ Listerin^fl/fl^ Lyz2^Cre^* mice were fed a WD for 16 weeks. (**A**) The measurement of BW and serum levels of triglycerides (mmol/L), cholesterol (mmol/L), HDL-C (mmol/L), and LDL (mmol/L). *n* = 8 per group. (**B**) Representative images and quantitation of aortic arch regions containing white plaques (yellow arrowheads). (**C**) En face Oil Red O staining and (**D**) quantitation of atherosclerotic plaques in the whole aorta. *n* = 8 per group. (**E**) H&E staining of representative aortic root sections and quantification of lesion area and necrotic core area. *n* = 8 per group. Scale bar: 200 μm. (**F**) Oil Red O–stained cross-section images and analysis of atherosclerotic plaques in the aortic root. *n* = 8 per group. Scale bar: 200 μm. Immunofluorescence staining for (**G**) CD68 and (**H**) ABCA1 in the aortic root. *n* = 8 per group. Scale bars: 200 μm (**G**) and 100 um (**H**). (**I**) Immunoblot images and quantitative analysis of ABCA1 in whole-aorta lysates from *ApoE^–/–^ Listerin^fl/fl^* and *ApoE^–/–^ Listerin^fl/fl^ Lyz2^Cre^* mice. *n* = 8 per group. Data are presented as the mean ± SD. The Shapiro-Wilk method was used to test normal distributions. Statistical analysis was performed using an unpaired, 2-tailed Student’s *t* test. **P < 0.01 and ***P < 0.001.

Then, we investigated the physiological role of Listerin in atherosclerosis. *Listerin^fl/fl^ Lyz2^Cre^* mice were crossed with *ApoE^–/–^* mice to generate *ApoE^–/–^ Listerin^fl/fl^ Lyz2^Cre^* mice. *ApoE^–/–^ Listerin^fl/fl^ Lyz2^Cre^* mice and *ApoE^–/–^ Listerin^fl/fl^* mice were fed a WD for 16 weeks and were then euthanized and evaluated for atherosclerosis. The results showed no significant difference in BW (Figure 7A and Supplemental Figure 6A) or serum triglyceride, cholesterol, HDL, or LDL levels (Figure 7A and Supplemental Figure 6A) between *ApoE^–/–^ Listerin^fl/fl^* and *ApoE^–/–^ Listerin^fl/fl^ Lyz2^Cre^* mice. However, *ApoE^–/–^ Listerin^fl/fl^ Lyz2^Cre^* mice exhibited many more lesions in the whole aorta and aortic root area than did *ApoE^–/–^ Listerin^fl/fl^* mice (Figure 7, B–D, and Supplemental Figure 6, B–D). Furthermore, the atherosclerotic lesion area and necrotic core area in the aortic root of *ApoE^–/–^ Listerin^fl/fl^ Lyz2^Cre^* mice were significantly increased compared with *ApoE^–/–^ Listerin^fl/fl^* mice (Figure 7E and Supplemental Figure 6E). We then performed a more detailed analysis of aortic root components. When compared with *ApoE^–/–^ Listerin^fl/fl^* mice, morphological analyses of the cross-sectional lesions showed that lipid accumulation and macrophage proportions increased in *ApoE^–/–^ Listerin^fl/fl^ Lyz2^Cre^* mice (Figure 7, F and G, and Supplemental Figure 6, F and G). We further examined ABCA1 expression levels in aortic lesions, and immunofluorescence of the aortic root and Western blotting of whole aorta lysates showed that ABCA1 levels were significantly decreased in *ApoE^–/–^ Listerin^fl/fl^ Lyz2^Cre^* mice compared with lysates from *ApoE^–/–^ Listerin^fl/fl^* mice (Figure 7, H and I, and Supplemental Figure 6, H and I). Taken together, these data show that Listerin deficiency in macrophages aggravates atherosclerosis development.

### Listerin overexpression ameliorates the development of atherosclerosis in vivo.

**Figure 8:**
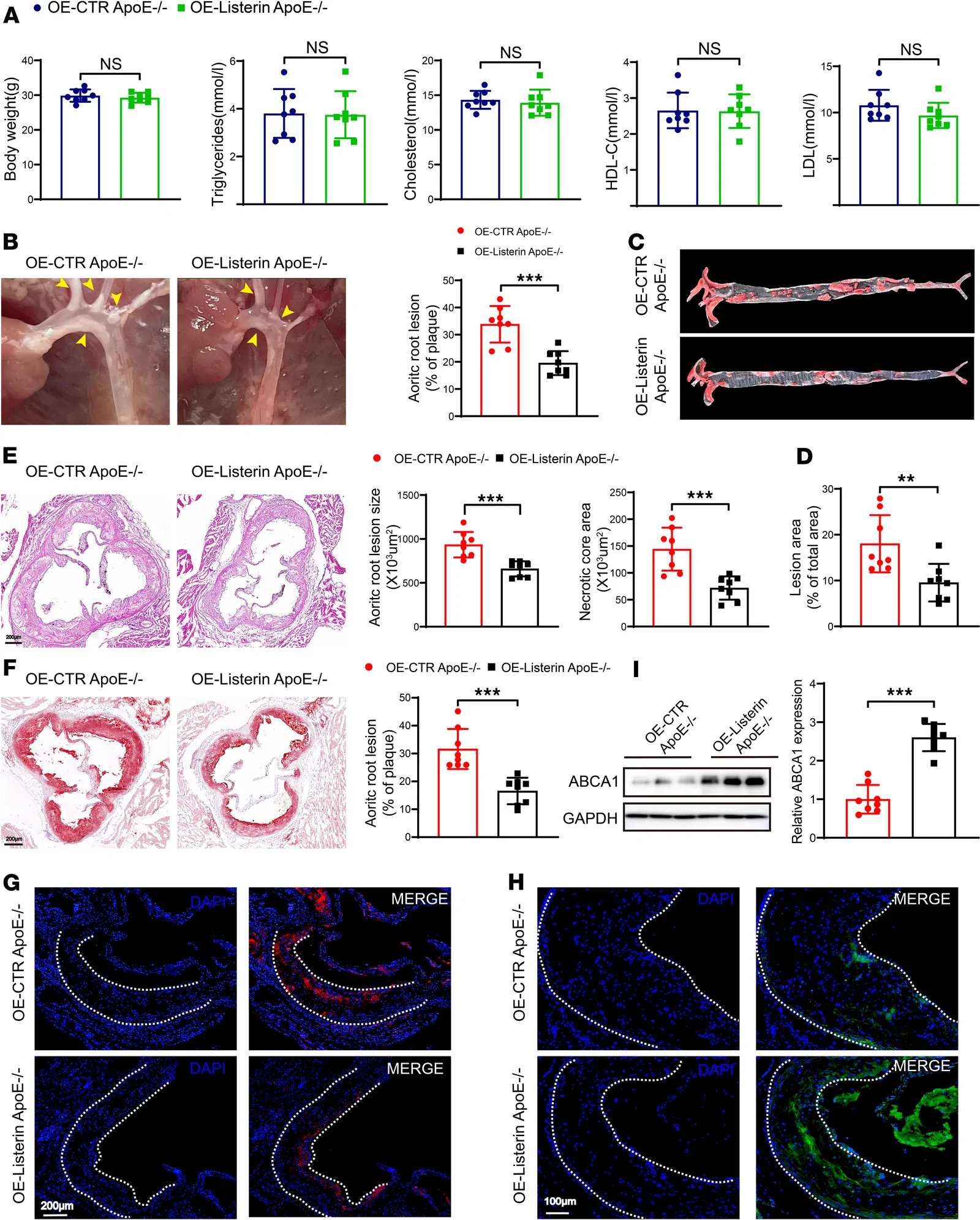
Listerin overexpression ameliorates the development of atherosclerosis in vivo. (**A**) Measurement of BW and serum levels of triglycerides (mmol/L), cholesterol (mmol/L), HDL-C (mmol/L), and LDL (mmol/L). *n* = 8 per group. (**B**) Representative images and quantitation of aortic arch regions containing white plaques (yellow arrowheads). (**C**) En face Oil Red O staining and (**D**) quantitation of atherosclerotic plaques in the whole aorta. *n* = 8 per group. (**E**) H&E staining of representative aortic root sections, quantification of lesions area and necrotic core area. *n* = 8 per group. Scale bar: 200 μm. (**F**) Oil Red O–stained cross section analysis of atherosclerotic plaques in the aortic root. *n* = 8 per group. Scale bar: 200 μm. Immunofluorescence staining for (**G**) CD68 and (**H**) ABCA1 in aortic root. *n* = 8 per group. Scale bars: 200 μm (**G**) and 100 μm (**H**). (**I**) Immunoblot images and quantitative analysis of ABCA1 in whole-aorta lysates from *OE-CTR ApoE^–/–^* and *OE-Listerin ApoE^–/–^* mice. *n* = 8 per group. Data are presented as the mean ± SD. The Shapiro-Wilk method was used to test normal distributions. For comparisons between the 2 groups, an unpaired, 2-tailed Student’s *t* test was used if the data were normally distributed, and the Mann-Whitney *U* test was performed if not. **P < 0.01 and ***P < 0.001.

To further verify the atherosclerotic regulatory function of Listerin, we constructed a Listerin-overexpressing adenovirus with the macrophage-specific promoter Lyz2 (OE-Listerin adenovirus).Both the control virus (OE-CTR) and the Listerin-overexpressing virus were injected into ApoE-/- mice via tail vein injection. The mice were fed a WD for 16 weeks to evaluate the atherosclerosis. Immunofluorescence results showed that the expression of Listerin in plaques of *OE-Listerin ApoE^–/–^* mice was markedly increased compared with that in plaques from control (*OE-CTR ApoE^–/–^*) mice (Supplemental Figure 7). In addition, there were no significant differences in BW or cholesterol, triglyceride, HDL, or LDL levels between *OE-CTR ApoE^–/–^* mice and *OE-Listerin ApoE^–/–^* mice (Figure 8A). Assessment of the en face lesion area and Oil Red O staining of aortas revealed that the atherosclerotic lesion area in the whole aorta of *OE-Listerin ApoE^–/–^* mice was significantly reduced compared with that of *OE-CTR* *ApoE^–/–^* mice (Figure 8, B–D). Furthermore, H&E and Oil Red O staining of aortic root lesions demonstrated that the lesion area, necrotic core area, and lipid deposition in *OE-Listerin ApoE^–/–^* mice were reduced compared with the control group (Figure 8, E and F). The immunofluorescence staining results for aortic root lesions showed that, compared with *OE-CTR* *ApoE^–/–^* mice, macrophage infiltration in *OE-Listerin ApoE^–/–^* mice was decreased (Figure 8G). Finally, we observed increased ABCA1 immunofluorescence staining in aortic root lesions from *OE-Listerin ApoE^–/–^* mice, which was consistent with the Western blot results for whole aorta lysates from these mice (Figure 8, H and I). Together, these data demonstrate that therapeutic overexpression of Listerin during atherosclerotic progression induced by a WD could dramatically reduce aortic lesion areas by promoting ABCA1 expression.

### Listerin regulates the progression of atherosclerosis through ABCA1.

To further investigate the molecular mechanism by which Listerin regulates atherosclerosis progression and clarify the role of ABCA1 in this process, we conducted adenovirus-mediated overexpression of ABCA1 or its Listerin-targeted ubiquitination site mutant (K1884R/K1957R) in macrophage-specific Listerin-KO mice to determine whether ABCA1 reconstitution could counteract the proatherogenic effects of Listerin deficiency. In a WD-induced atherosclerosis model, no significant differences in BW or cholesterol, triglyceride, HDL, or LDL levels were observed among *ApoE^–/–^ Listerin^fl/fl^*, *ApoE^–/–^ Listerin^fl/fl^ Lyz2^Cre^*, *ApoE^–/–^ Listerin^fl/fl^ Lyz2^Cre^+ABCA1*, or *ApoE^–/–^ Listerin^fl/fl^ Lyz2^Cre^+ABCA1* (mut) groups (Supplemental Figure 9A). However, en face aortic lesion analysis (Supplemental Figure 9B) and Oil Red O staining (Supplemental Figure 9C) demonstrated that Listerin KO exacerbated AS progression, whereas overexpression of ABCA1 or its ubiquitination site mutant reversed the proatherogenic effects caused by Listerin deficiency, restoring plaque burden to control levels. Western blotting further confirmed reduced ABCA1 protein expression in Listerin-KO plaques, which was rescued by overexpression of either WT ABCA1 or its ubiquitination-defective mutant (Supplemental Figure 9D).

Finally, we generated a macrophage-specific, Listerin-overexpressing adenovirus (OE-Listerin) and an ABCA1-knockdown adeno-associated virus (shABCA1-AAV). These viruses, alongside control viruses, were delivered to *ApoE^–/–^* mice fed a WD to establish an atherosclerosis model. Immunofluorescence confirmed markedly downregulation of ABCA1 expression in atherosclerotic plaques and macrophages following shABCA1-AAV administration (Supplemental Figure 8). No differences in BW or cholesterol, triglyceride, HDL, or LDL levels were observed among the *OE-Ctrl ApoE^–/–^*, *OE-Listerin ApoE^–/–^*, or sh*ABCA1+OE-Listerin* *ApoE^–/–^* groups (Supplemental Figure 9E). Evaluation of the en face aortic lesion area (Supplemental Figure 9F) and Oil Red O staining (Supplemental Figure 9G) revealed a reduced plaque area in Listerin-overexpressing macrophages (*OE-Listerin ApoE^–/–^* mice) and abrogation of Listerin’s protective effect upon concurrent ABCA1 knockdown (sh*ABCA1+OE-Listerin* *ApoE^–/–^* mice). Finally, Western blot analysis of ABCA1 revealed increased ABCA1 expression in OE-Listerin lesions and restoration of ABCA1 to control levels in *shABCA1+OE-Listerin* mice (Supplemental Figure 9H). These data collectively demonstrate that therapeutic Listerin overexpression attenuated the aortic lesion area in WD-induced atherosclerosis by ubiquitinating ABCA1 at lysine residues K1884 and K1957 to stabilize and upregulate ABCA1 expression, thereby establishing ABCA1 as the critical mediator of Listerin’s atheroprotective effects.

## Discussion

ABCA1 is a pivotal cholesterol transporter on macrophages that mediates cholesterol efflux to extracellular acceptors (ApoA1), and accumulating evidence has shown that macrophage ABCA1 alone plays an important role in atherogenesis. Macrophage ABCA1 degradation leads to lipid deposition and foam formation in macrophages, promoting the development of atherosclerosis (47–51). Targeting macrophage ABCA1 has emerged as a viable strategy for attenuating atherosclerotic progression. In addition, it has been reported that advanced glycation end-product (AGE) albumin diminishes ABCA1 by accelerating its ubiquitination and degradation through the proteasome and lysosome systems (38). Forced expression of COP9 signalosome subunit 3 (CSN3) inhibits thrombin-induced ABCA1 ubiquitination and degradation (48). Cell cholesterol loading also inhibits the ubiquitination and proteasomal degradation of ABCA1 and ABCG1 (52). Aside from protein degradation, ubiquitination is also closely related to protein stability or trafficking of ABCA1 (22, 53–55), which plays an important role in atherosclerosis. In the human hepatoma cell line HepG2, but not in PMs, the long form of Pim1 (Pim1L) interacts with cell-surface-resident ABCA1 (csABCA1), thereby protecting it from ubiquitination and subsequent lysosomal degradation via its phosphorylation function (54). In another study, the authors found that csABCA1 degradation is inhibited by overexpression of a dominant-negative form of ubiquitin. Moreover, disruption of the ESCRT pathway, a dominant mechanism for ubiquitination-mediated lysosomal degradation, significantly delays the degradation of cell-surface-resident ABCA1 (22).

The above studies showed that, with the help of ubiquitination modifications, ABCA1 becomes unstable and is easily degraded through the proteasome or lysosome pathway (38), and the ESCRT pathway plays an important role in the stability and trafficking of ABCA1 (22). However, some key scientific questions regarding ABCA1 ubiquitin modification remain unanswered. First, an E3 ligase that directly binds to ABCA1 has not yet been identified. Second, many types of protein ubiquitination exist, and different types of ubiquitin modifications have different effects on protein function. For example, K48-linked polyubiquitination usually regulates protein degradation, and K63-linked polyubiquitination might be related to protein translocation and signaling activation (56). To date, no detailed studies have been performed on the different types of ubiquitin modifications of ABCA1. Third, the ESCRT lysosome is an important pathway that regulates ABCA1 protein stability, but the mechanism of ubiquitination is still unclear. In our study, we found that Listerin was essential for the degradation of cell-surface-resident ABCA1 through the ESCRT system, because ABCA1 protein levels were restored by either HRS-specific siRNA or the ESCRT inhibitor DBeQ in Listerin-deficient PMs. Furthermore, our research shows that Listerin, acting as an E3 ubiquitin ligase, bound ABCA1 and promoted K63-linked polyubiquitination of ABCA1 at lysine sites K1884 and K1957, which inhibited its translocation from the cytomembrane and degradation through the ESCRT/lysosome pathway.

Several studies have demonstrated that LTN1 (a yeast homolog of Listerin) is a component of the RQC complex, especially in yeast and bacteria, and that LTN1mediates the ubiquitination and extraction of incompletely synthesized nascent chains for proteasomal degradation with its cofactor Rqc2 (NEMF in mammals) (43–45). Unlike Rqc2/NEMF, the core component of the RQC system, recent studies have shown that Listerin may play other roles independent of the RQC system in more complex mammalian cells (49, 57–59). For instance, Listerin negatively regulates the cyclic GMP-AMP synthase–mediated (cGAS-mediated) immune response by facilitating the degradation of cGAS protein via the ESCRT pathway (49). Another study reports that Listerin also negatively modulates retinoic acid-inducible gene I (RIG-I)-like receptors (RLR)-mediated antiviral innate immunity against RNA viruses through the ESCRT pathway (58). While the cGAS/stimulator of IFN genes (cGAS/STING) signaling pathway has been well documented in mediating inflammatory responses during atherosclerosis progression (60), emerging evidence suggests potential crosstalk between Listerin and cGAS in viral infection contexts. Our experiments revealed no detectable changes in cGAS expression within atherosclerotic plaques of macrophage-specific Listerin-KO or Listerin-overexpressing mice compared with control mice (data not shown). This observation suggests that, unlike its role in acute infectious diseases, Listerin may regulate alternative targets beyond the cGAS/STING axis in chronic pathologies like atherosclerosis. These findings highlight the idea that Listerin engages distinct molecular targets and modulates divergent biological processes depending on cellular context and pathological milieu, thereby revealing a unique mechanistic framework in atherosclerosis pathogenesis. Our study found that Listerin targeted ABCA1 for K63-linked polyubiquitination independent of the RQC system and also further supports the idea that Listerin might play important roles in mammals.

As mentioned above, E3 ubiquitin ligase LTN1 (a yeast homolog of Listerin) has attracted increasing attention, with a focus on yeast and in vitro translation systems (43–45). Exploration of the role of Listerin in mammalian physiology and disease is necessary. In our study, Listerin conditional–KO mice were generated, and we found that *ApoE^–/–^ Listerin^fl/fl^ Lyz2^Cre^* mice had more severe plaque progression than did *ApoE^–/–^ Listerin^fl/fl^* mice. Conversely, macrophage-specific overexpression of Listerin attenuated plaque formation compared with control mice. Critically, Listerin’s regulation of atherosclerotic plaques is dependent on ABCA1. Furthermore, we observed no significant differences in BW or serum lipid profiles between the groups. The potential reason for unchanged murine lipid profiles following Listerin intervention is that, unlike hepatocytes, in which cholesterol metabolism directly regulates systemic serum lipid levels, sparsely distributed macrophages predominantly influence local vascular cholesterol accumulation. These data demonstrate the physiological phenomenon in mice that loss of Listerin in macrophages aggravates atherosclerosis progression. It has been reported that mutations in Listerin cause neurodegeneration in mice (61), but the mechanistic details were not elucidated. ApoE lipidation, which was controlled by ABCA1 activity, was reported to play a central role in β-amyloid (Aβ) accumulation and Alzheimer’s disease (AD) pathology. Enhancing ABCA1 recycling to the membrane could restore ABCA1 activity and enhance Aβ degradation (53). In our study, we found that Listerin could combine with ABCA1 and promote K63-linked polyubiquitination of ABCA1, which inhibited its translocation from the cytomembrane and its degradation. Our data might partly explain why Listerin mutations aggravate neurodegeneration in mice.

In summary, our study demonstrates that the macrophage Listerin has an important effect on the pathogenesis of atherosclerosis, an effect different from that seen in yeast and bacteria, and reveals another regulatory mechanism of ABCA1. Listerin catalyzed K63-linked polyubiquitination of ABCA1 at lysine sites K1884 and K1957 and inhibited its degradation through the ESCRT lysosome pathway, which further promoted the cholesterol efflux of macrophages, inhibited foam cell formation, and ameliorated atherosclerosis development. Since macrophages play a key role in atherosclerosis progression, macrophage-mediated proatherosclerotic processes are important targets for developing diagnostic imaging methods and therapies for atherosclerosis (3). Nanotherapy targeting macrophage Listerin may be a meaningful therapeutic strategy for ameliorating atherosclerosis by activating the macrophage cholesterol efflux receptor ABCA1.

## Methods

All data supporting the findings of this study can be made available to other researchers for reproducing the results or replicating the procedures. Further details on the materials and methods are provided in the supplemental materials.

### Sex as a biological variable.

Our study examined both males and females in mouse experimental models, human biospecimen studies, and human PBMCs, and similar findings are reported for both sexes; therefore, sex was not considered as a biological variable.

### Animals.

Listerin-deficient mice were generated by Beijing Biocytogen using the CRISPR/Cas9 system, and genotyping of mice was performed using the following primers: forward, 5′-GGAGTTACA GCTGGGAGTTGTCGTG-3′ and reverse, 5′-GCTCAGCAATATCACAACGCTGCAT-3′. The mice were crossed with Lyz2^Cre^ mice [B6.129P2-Lyz2tm1(Cre)Ifo/J, The Jackson Laboratory] to obtain Listerin^fl/fl^ Lyz2^Cre^ mice. These mice were then backcrossed onto the C57BL/6 background (GemPharmatech, N000013). Furthermore, Listerin^fl/fl^ Lyz2^Cre^ mice were crossed with ApoE^–/–^ (B6.129P2- Apoetm1Unc/J, The Jackson Laboratory) mice to obtain ApoE^–/–^ Listerin^fl/fl^ Lyz2^Cre^ mice, and these mice were also backcrossed onto the C57BL/6 background (GemPharmatech, N000013). To generate an atherosclerosis animal model, mice were fed a WD for 16 weeks. None of the mice exhibited a difference in phenotype, and both male and female mice were used. Based on our preliminary experimental results, we set the significance level (α) at 0.05 and power (1-β) at 80% to confirm the sample size, and 8–10 animals were analyzed in each group. Animals were grouped based on genotype. Mice were subsequently subjected to high-fat diet administration in a blinded manner to eliminate operator bias in atherosclerotic plaque progression assessment. Animals that were sick or died before euthanasia were not included in the final analysis.

### Human coronary artery samples.

Human coronary atherosclerotic plaques were obtained from autopsy specimens from 6 male body donors with coronary heart disease after sudden coronary death, and the bodies were provided by the Red Cross Society of Shandong Province in China. Baseline characteristics of the human specimens used in this study are listed in Supplemental Table 3. The coronary arteries were embedded with optimal cutting temperature compound (OCT) (Sakura Finetek) and cut into 7 μm thick cross-sections for histopathological staining.

### Cell culture.

HEK293T cells, HeLa cells, and THP-1 cells, and RAW264.7 macrophages were obtained from KeyGene BioTech. Primary peritoneal macrophages were extracted from Listerin^fl/fl^ Lyz2^Cre^ mice and their control littermates or C57BL/6J WT mice after i.p. injection of 1 mL 6% sterile starch, and all of these cells were cultured in DMEM containing 10% FBS and 1% penicillin-streptomycin at 37°C.

### Analysis of atherosclerotic plaques.

After 16 weeks of WD feeding, the mice were fasted for 12 hours and then euthanized using a single dose of pentobarbital (150 mg/kg, i.p.). Blood was drawn from the left ventricle and perfused with cold saline, and the heart and whole aorta were removed and fixed with 4% paraformaldehyde. For analysis of en face aorta, the paraformaldehyde was washed away with saline, the surrounding adipose tissue was removed and examined under a microscope, and the aorta was cut longitudinally for Oil Red O staining. For aortic root atherosclerotic lesions, the base of the heart and the root of the ascending aorta were dissected, embedded with OCT, and sequential cross-sections at 7 μm distance per section from the origin of the aortic valves to the ascending aorta were collected. The frozen sections were stained with H&E or Oil Red O, or were prepared for immunofluorescence staining, complying with the manufacturer’s instructions. Then, the stained slides were observed under a microscope. Researchers were blinded to the mouse genotype for the measurement of atherosclerotic plaque.

### Statistics.

For immunoblots, protein band intensities were quantified using ImageJ, version 1.52a (NIH) and normalized to the corresponding GAPDH levels. All data are expressed as the mean ± SD. Statistical analyses were performed using GraphPad Prism 8 (GraphPad Software), with each experiment independently repeated at least 3 times. Normally distributed data were analyzed by unpaired, 2-tailed Student’s *t* tests, whereas non-normally distributed data were assessed using the Mann-Whitney *U* test. For 1-variable comparisons across multiple groups, 1-way ANOVA with Dunnett’s post hoc test was applied. Multivariate comparisons were conducted with a 2-way ANOVA followed by Šidák’s multiple-comparison test. Non-Gaussian distributed single-variable datasets were subjected to Kruskal-Wallis testing with Dunnett’s post hoc analysis for intergroup comparisons. *P* of less than 0.05 were considered significant.

### Study approval.

All experiments involving humans were reviewed and approved by the Ethics Committee of Qilu Hospital of Shandong University (no. KYLL 2022(ZM)-427), and written, informed consent was obtained from all participants. All experiments using mice were conducted in accordance with the NIH’s *Guide for the Care and Use of Laboratory Animals* (National Academies Press, 2011) and approved by the Ethics Committee of Qilu Hospital of Shandong University [no. KYLL 2022(ZM)-427].

### Data availability.

RNA-Seq data were deposited in the NCBI’s Sequence Read Archive (SRA) database under accession number PRJNA1269750. Proteomics data are deposited in ProteomeXchange under accession numbers PXD064532 and PXD064538.

## Author contributions

MZ, CZ, and CG conceived the study, directed the research, and supervised all experimental work. LC, JZ, and LY performed most experiments, collected all the data, participated in results analysis, visualized data in publication-ready figures, and wrote the manuscript. WY, WQ, RR, YL, YH, and YC contributed to animal experiments and conducted laser confocal immunofluorescence detection. QL, XW, WS, and YZ participated in project discussions and data analysis. BL performed clinical blood sample collection. ZZ obtained atherosclerotic tissue specimens. Funding acquisition was the responsibility of CZ and MZ. All authors have read and approved the article. LC, JZ, and LY are co–first authors. The order of co–first authors was determined by their relative contributions.

## Supplementary Material

Supplemental data

Unedited blot and gel images

Supporting data values

## Figures and Tables

**Table 1:**
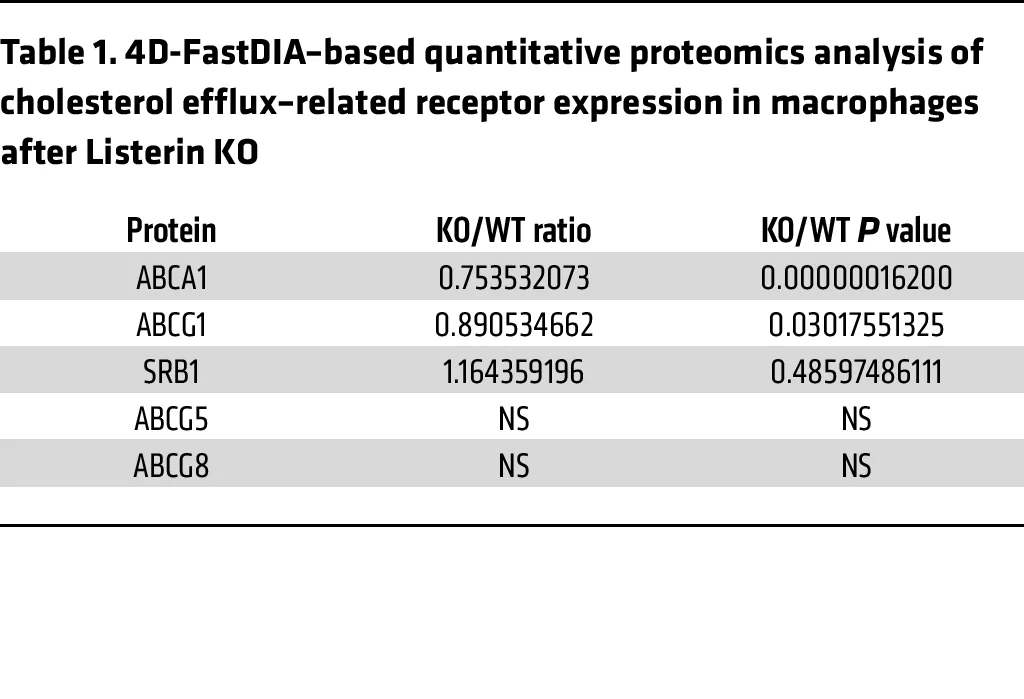
4D-FastDIA–based quantitative proteomics analysis of cholesterol efflux–related receptor expression in macrophages after Listerin KO

## Version 1 06/17/2025

In-Press Preview

## Version 2 08/15/2025

Electronic publication

## Version 3 09/15/2026

Corrected data in Figure 2K
