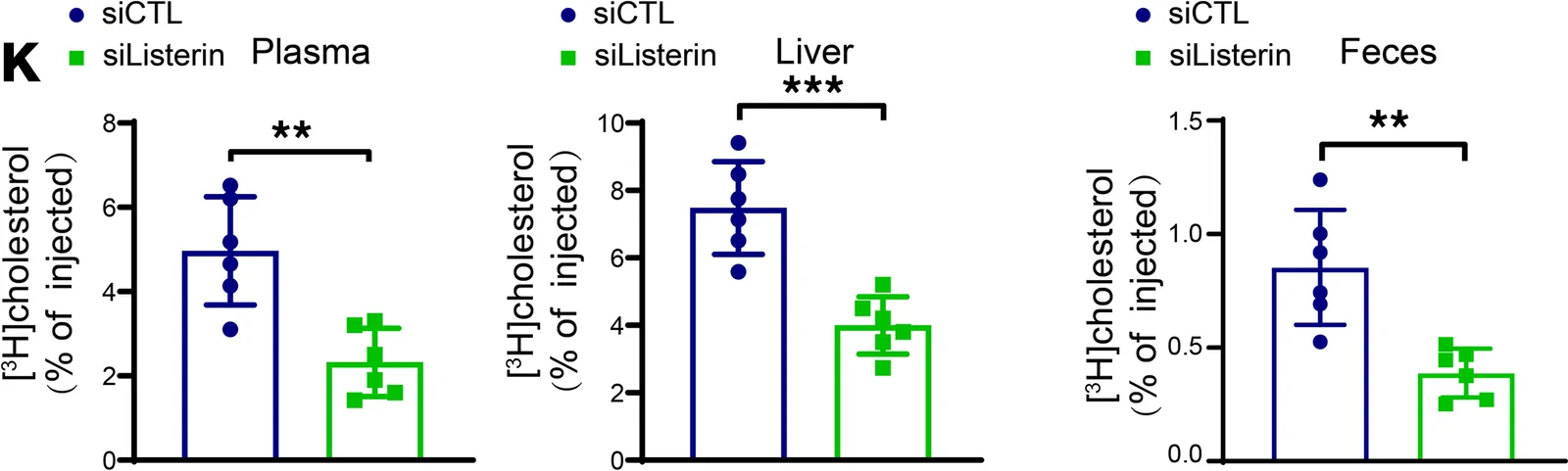# Corrigendum to E3 ubiquitin ligase Listerin regulates macrophage cholesterol efflux and atherosclerosis by targeting ABCA1

**DOI:** 10.1172/JCI211585

**Published:** 2026-09-15

**Authors:** Lei Cao, Jie Zhang, Liwen Yu, Wei Yang, Wenqian Qi, Ruiqing Ren, Yapeng Liu, Yonghao Hou, Yu Cao, Qian Li, Xiaohong Wang, Zhengguo Zhang, Bo Li, Wenhai Sui, Yun Zhang, Chengjiang Gao, Cheng Zhang, Meng Zhang

Original citation: *J Clin Invest*. 2025;135(16):e186509. https://doi.org/10.1172/JCI186509

Citation for this corrigendum: *J Clin Invest*. 2026;136(18):e211585. https://doi.org/10.1172/JCI211585

After publication of this article, the authors became aware of the following error: The siCTL and siListerin data underlying the right panel (“Feces”) of Figure 2K were incorrectly derived from the same control group samples and represent siCTL values with and without supernatant volume correction, respectively. Using the raw data from the original liquid scintillation counter animal fecal matter records, we recalculated the data for each sample with the following formula: excretion rate = (fecal radioactivity − blank control) × 2/(total injected radioactivity/mouse − blank control) × 100%. The correct figure panel is shown below. The HTML and PDF versions of the article, including Supporting data values, have been updated online.

The authors regret the error.

## Figures and Tables

**Figure 2K:**