# Supplementary material for: E3 ubiquitin ligase Listerin regulates macrophage cholesterol efflux and atherosclerosis by targeting ABCA1
Source: J Clin Invest. 2025 Jun 17;135(16):e186509. doi: 10.1172/JCI186509 (PMC12352907; doi:10.1172/JCI186509)
Supplement: Supplemental data [file jci-135-186509-s078.pdf]

Supplemental Materials

Supplementary Table S1. Antibodies and manufacturers

Antibody	Company	Catalog No.	Concentrations
Listerin	Abcam	Ab104375	WB: 1:1000, IF:1:100, IP:1:400
ABCA1	Abcam	Ab7360	WB: 1:1000
ABCA1	Abcam	Ab18180	WB: 1:1000, IF; 1:100
ABCA1	Cell Signaling Technology	#96292S	WB: 1:1000, IP1:50
GAPDH	Cell Signaling Technology	#2118	WB: 1:1000
ABCG1	Abcam	Ab52617	WB: 1:1000
SR-A1	Abcam	Ab151707	WB: 1:1000
SR-B1	Abcam	Ab52629	WB: 1:1000
CD36	Abcam	Ab133625	WB: 1:1000
CD68	Abcam	Ab125212	WB: 1:1000
CD68	Novus	NBP2-33337	IF： 1:200
MOMA-2	Abcam	Ab33415	IF: 1:200
ATG5	Cell Signaling Technology	12994	WB: 1:1000
HRS	Santa Cruz Biotechnology	sc-271455	WB: 1:1000
Na, K-ATPase	Cell Signaling Technology	#3010	WB: 1:1000
NEMF	Proteintech	11840-1-AP	WB: 1: 500
Ubquitin	Abcam	Ab134953	WB: 1:1000
Ubiquitin (linkage-	Abcam	Ab140601	WB: 1:1000

specific K48)			
Ubiquitin (linkage-specific K63)	Abcam	Ab179434	WB: 1:1000
HA	ORIGENE	TA180128	WB: 1:1000
Myc	Cell Signaling Technology	#2278	WB: 1:1000
Flag	Sigma-Aldrich	F1804	WB: 1:1000
GFP	Cell Signaling Technology	#2956	WB: 1:1000

Supplementary Table S2. Primer sequence of genes

Genes	Primer type	Sequence 5'-3
ABCA1	Forward	GACCCGCGCAACCACA
	Reverse	CTCTGTGGCTGGTCATTAACTGT
ABCG1	Forward	GGTGTTGGGTGGACAAACCT
	Reverse	TGCGGACATGGATGGAATGG
SR-A1	Forward	CAGAGTCCGTGAATCTACAG
	Reverse	GGTCGTTGGTGATGTTGT
SR-B1	Forward	CCGACCCTGTGTTGTCAGAA
	Reverse	CCACAGCAACGGCAGAACTA
CD36	Forward	AGGTCTATCTACGCTGTGT
	Reverse	CAATGGTTGTCTGGATTCTG
CD68	Forward	TGGCGGTGGAATACAATG
	Reverse	GATGAGAGGCAGCAAGAG
β-actin	Forward	CCACACCCGCCACCAGTTCG

	Reverse	TACAGCCCGGGGAGCATCGT
Listerin	Forward	ACCTGGCGTGCTCGGTCTA
	Reverse	TTCCCACTTTCCACTGTTATTGA

Supplementary Table S3. Clinical features of patients with coronary atherosclerotic plaques

	No	Age (years)	Gender	Body weight (Kg)	Blood lipids(mmol/L)			
					LDL-C	HDL-C	TG	TC
Early leisions	1	64	Male	65	0.45	0.56	0.49	1.21
	2	51	Male	72	3.5	0.7	2.15	4.9
	3	64	Male	62	2.21	0.43	1.79	3
	4	49	Male	65	1.84	0.79	0.88	3.05
	5	23	Male	70	2.07	1.05	0.62	3.35
	6	65	Female	60	1.61	0.54	1.14	2.5
Advanced leisions	1	66	Male	63	3.49	0.73	2.91	5.75
	2	57	Male	71	0.95	1.51	3.69	4.36
	3	38	Male	45	2.76	0.67	1.12	4.05
	4	64	Female	75	2.93	0.65	2.22	4.65
	5	44	Male	65	2.37	0.52	1.53	3.46
	6	40	Male	80	2.53	0.61	0.81	3.3

LDL-C, low density lipoprotein cholesterol; HDL-C, high density lipoprotein cholesterol; TG, total triglycerides; TC, total cholesterol.

Supplementary Table S4. Clinical features of human participants

Characteristic	Control group (n=19)	ASCVD group (n=27)	P values
Ages (years)	60.26±7.69	63.19±11.83	0.3507
Male, n (%)	10(52.6)	18(66.6)	0.337
Smoking (n, %)	3(15.8)	9(33.3)	0.115
BMI (kg/m^2)	25.6±3.03	26.6±4.0	0.1919
TG (mmol/L)	1.54±0.89	1.52±0.65	0.7529
TC (mmol/L)	4.8±0.71	3.71±0.91	<0.001
HDL (mmol/L)	1.22±0.28	1.12±0.24	0.1994
LDL-C (mmol/L)	3.04±0.69	2.12±0.83	<0.001

ASCVD, Atherosclerotic cardiovascular disease; LDL-C, low density lipoprotein cholesterol; HDL-C, high density lipoprotein cholesterol; TG, total triglycerides; TC, total cholesterol.

Expanded Materials and Methods

Reagents and Antibodies

Human Oxidized LDL was purchased from Peking Union-Biololgy, Human Dil-ox-LDL was purchased from Yiyuan biotechnology, Cycloheximide (CHX), MG132, Calpeptin, Bafilomycin A1, Wortmannin, DBeQ were purchased from MCE(USA), Chloroquine, 3-MA were from Sigma-Aldrich (USA), ApoA1 was purchased from R&D, NBD-cholesterol were purchased from ThermoFisher, Protein A/G agarose was

purchased from Santa Cruz Biotechnology (USA). Erythrodiol was purchased from MCE. Primary antibodies used for Flow cytometry were Listerin (produced by ABclonal, Project No. WG-059009; immunogen: MGGKNKQRTKGNLRPSNSGRAAELLAKEQGTVPGFIGFGTSQSDLGYVPA-C) and ABCA1 (Novus Cat# NB400-105AF488). Others were listed in Supplementary Table S1.

Human PBMCs

The human study protocol was approved by the Ethical Committee of Qilu Hospital of Shandong University (No. KYLL 2022(ZM)-427). All participants gave informed consent to blood sample donation and use of their clinical data for research purposes. PBMCs were isolated from blood collected from 27 atherosclerotic cardiovascular disease (ASCVD) patients diagnosed through clinical evaluation and coronary angiography. Exclusion criteria were inability to give informed consent, age less than 18 years old, active cancer treated with chemotherapy or radiation, taking immune-suppressive drugs, and pregnancy. Nineteen age-matched healthy controls were selected from individuals undergoing routine health screening populations, with exclusion criteria including a history of coronary artery disease (CAD), peripheral atherosclerotic vascular disease, cerebrovascular stroke, or myocardial infarction. PBMCs isolation protocols using density gradient centrifugation were uniformly implemented for both groups. Baseline characteristics of the human specimens used in the study are listed in Supplementary Table S4.

Isolation of primary peritoneal macrophages and treatment

Mice were injected intraperitoneally with 1 ml of 6% sterile starch. After 3 days, the mice were euthanized and then sterilized with 75% ethanol for 5min. The skin was cut open to expose the peritoneum and the abdominal cavity was irrigated 3 times with 30ml DMEM, then the cell suspension was centrifuged at 800 rpm for 5 min. The supernatant was discarded and cell precipitate was suspended by DMEM (Hyclone, USA) containing 10% FBS (Sigma),1%penicillin-streptomycin (Gibco, USA) at 37 ℃. Use these reagents to treat macrophages: Human Oxidized LDL (Peking Union-Biololgy, China), cycloheximide (MCE, USA).

Analysis of foam cell formation

Primary peritoneal macrophages were incubated with 50μg/ml Oxidized LDL (ox-LDL) for 24 hours, The cells were washed 3 times with PBS and then fixed with 4% paraformaldehyde, washing paraformaldehyde with PBS, then the cells were stained with 0.5% Oil red O for 1 hour, decolorized with 60% isopropyl alcohol for several seconds, and the nucleus was stained with hematoxylin after washing, and sealed with glycerin jelly.

Analysis of Dil-ox-LDL uptake

Primary peritoneal macrophages were incubated with 40μg/ml Dil-ox-LDL for 4 hours, The cells were washed 3 times with PBS and then fixed with 4% paraformaldehyde, and a fluorescence microscope was used to measure the fluorescence intensity of the cells. The images should be exposed at the same time.

Analysis of Cholesterol efflux

PMs were incubated with 1μg/ml NBD-cholesterol (ThermoFisher, USA) for 6 h. After

cholesterol loading, the cells were washed and equilibrated for 2 h, then incubated with Dulbecco's Modified Eagle Medium containing no phenol red (Gibco, 21063029) with 0.2% BSA (Biofroxx, 9048-46-8) and APOA1(10μg/ml) for 6 h. Control wells were treated with 0.2% BSA to measure the background. collected the medium, and the cells were lysed at 37°C with 0.3 M NaOH solution for 15 min. Used microplate spectrophotometer to measure the fluorescence intensity of medium and cellular lysate. Calculation of cholesterol efflux ratio: The percentage of medium fluorescence intensity relative to total fluorescence intensity (medium and lysate).

ABCA1, HRS, ATG5 and NEMF KO THP-1 cells

THP-1 knockout cells were generated using the CRISPR/Cas9 system. The plasmid driven sgRNA of ABCA1, HRS, ATG5 or NEMF was electroporated into THP-1 cells (5 μg plasmid were mixed with 250 μl cell suspension for 10×10^6 cells/ml in RPMI-1640) and electroporated at 950 μF and 250 V. Cells were FACS sorted after 24 h electroporation. Positive cells were diluted under limiting conditions and plated in 96-well plates to obtain single cell clones. The genotype of THP-1 clones was analyzed by deep sequencing.

In Vivo Reverse Cholesterol Transport Assay (RCT)

RAW 264.7 macrophages were transiently transfected with either: (a) negative control siRNA (NC) or Listerin-targeting siRNA (siListerin); or (b) empty vector (Vector), ABCA1-expressing plasmid, or ABCA1-K1884/K1957-expressing plasmid. Transfected cells were cholesterol-loaded and radiolabeled via 48-h incubation with 5 μCi/mL [^{3}H]cholesterol and 50 μg/mL acetylated LDL. Following washing, cells

underwent 24h incubation in serum-free medium to enable cholesterol efflux, were harvested, and resuspended in 0.15 M sterile saline. For in vivo reverse cholesterol transport (RCT) assessment: Listerin knockdown groups received intraperitoneal injections of either NC-transfected macrophages (2.96×10^6 cells, 4.63×10^6 cpm/mouse) or siListerin-transfected macrophages (3.01×10^6 cells, 4.71×10^6 cpm/mouse); ABCA1 expression groups received Vector-transfected (2.55×10^6 cells, 4.31×10^6 cpm/mouse), ABCA1-transfected (2.53×10^6 cells, 4.28×10^6 cpm/mouse), or ABCA1-K1884/K1957-transfected macrophages (2.48×10^6 cells, 4.27×10^6 cpm/mouse). All injections utilized male C57BL/6 mice (n=6 per group). After 48 h, mice were euthanized with collection of livers and feces. Tissue lipids were extracted using hexane/isopropanol (3:2, v/v), dried overnight, solubilized, and quantified by liquid scintillation counting. RCT efficiency to plasma, liver, and feces was calculated as percentage recovery of total injected radioactivity.

Flow Cytometry Analysis

Primary peritoneal macrophages were isolated and prepared as a single-cell suspension. Cells were divided into three experimental groups: unstained control, isotype control, and test sample. For the test sample, cells were incubated with an ABCA1-specific antibody (diluted according to the manufacturer's protocol) and gently mixed, followed by incubation at 4°C for 30 minutes in the dark. The unstained control received no antibody, while the isotype control was treated with an equivalent concentration of isotype-matched immunoglobulin. After incubation, cells were washed twice with PBS and resuspended in 400 μL PBS for flow cytometry analysis using a BD FACSCanto™.

Data acquisition and analysis were performed with FlowJo v10.8 to quantify ABCA1 surface expression.

De novo Lipid Synthesis Assay

Lipid biosynthesis was assessed by culture of PMs (2 $\times 10^6$) in complete medium containing 4 μCi/mL Acetic acid [3^H] (PerkinElmer, Waltham, MA, United States), and stimulated as described in the figure legends. After 4h incubation time, the buffer was aspirated, and cells were washed with cold PBS. The lipid fraction was extracted using Folch's extraction method. The extracted lipids were carefully transferred to scintillation vials and mixed with Opti-Fluorscintillation liquid, and total radioactivity incorporated in lipids was determined by liquid scintillation counting.

Quantitative proteomics analysis

The 4D-FastDIA-based quantitative proteomic analysis of PMs isolated from $Listerin^{fl/fl}$ and $Listerin^{fl/fl}Lyz2^{cre}$ mice was carried out by Jingjie PTM Biolabs Inc. (Hangzhou, China). Samples were sonicated three times on ice using a high-intensity ultrasonic processor (Scientz) in lysis buffer (8 M urea, 1% protease inhibitor cocktail). The remaining debris was removed by centrifugation at 12,000*g* at 4 °C for 10 min. Finally, the supernatant was collected, and the protein concentration was determined with a BCA kit according to the manufacturer's

scRNA-seq data pre-processing

FASTQ files of atherosclerotic plaques in mice with athero-prone backgrounds (Ldlr-/-) were obtained from the NCBI Gene Expression Omnibus (GEO) database under the accession number GSE155513. Two samples were obtained for each group at the

different timepoint of WD feeding (0, 8, 16, 26 weeks). Sample demultiplexing, barcode processing and single-cell 3'gene counting by using the Cell Ranger pipeline (https://support.10xgenomics.com/single-cell-geneexpression/software/pipelines/latest/what-is-cell-ranger,version5.0.1) and scRNA-seq data were aligned to Ensembl genome GRCm38 reference genome.

Dimensionality reduction and clustering

The Cell Ranger output was loaded into Seurat (version 4.3.0) be used to Dimensional reduction, clustering, and analysis of scRNA-seq data. All genes expressed in less than three cells were removed, number of genes expressed per cell > 200 as low and <4500 as high cut-off. The percent of mitochondrial-DNA derived gene-expression was less than 15%. To remove the batch effect, we performed the data integration and made the batch correction with Harmony. To visualize the data, we further reduced the dimensionality of all cells using Seurat and used UMAP to project the cells into 2D space. The LogNormalize method of the "Normalization" function of the Seurat software was used to calculated the expression value of genes. PCA (Principal component analysis) analysis was performed using the normalized expression value, within all the PCs, the top 10 PCs were used to do clustering and UMAP analysis. To find clusters, selecting weighted Shared Nearest Neighbor (SNN) graph-based clustering method. Marker genes for each cluster were identified with the Wilcoxon rank-sum test with default parameters via the FindAllMarkers function in Seurat. This selects markers genes which are expressed in more than 10% of the cells in a cluster and average log (Fold Change) of greater than 0.25.

Adenovirus infection

Adenoviruses of Listerin, Listerin-ΔRing, and Listerin-C/A were constructed in BioSune Biotechnology (Shanghai, China). To overexpress Listerin, Listerin-ΔRing, and Listerin-C/A, primary peritoneal macrophages of mice were cultured in 6-well plates, in the presence of 5μg/mL polybrene, and the cells were transfected with MOI=600 for 12 h and then cultured in fresh DMEM medium for 48 h.

Lysosome isolation and extraction

Lysosomes were isolated and extracted using the Minute™ Lysosome Isolation Kit (LY-034, Invent Biotechnologies, Plymouth, MN, USA). Briefly, cells were washed with pre-chilled PBS. Subsequently, 500 μl of Buffer A was added to the cell pellet and the mixture was incubated on ice for 5–10 minutes. Following incubation, the mixture was centrifuged at 16,000×g for 30 seconds. The resulting pellet was resuspended in Buffer A and transferred to the provided collection tubes. The suspension was then centrifuged at 2,000×g for 3 minutes to pellet cell nuclei, large debris, and any intact cells. The supernatant was carefully transferred to a new tube and centrifuged at 11,000×g for 15 minutes at 4°C. After discarding the supernatant, the pellet was resuspended in 200 μl of Buffer A, vortexed thoroughly, and centrifuged again at 2,000×g for 4 minutes. The supernatant from this step was transferred to a new tube, mixed with 100 μl of Buffer B, and vortexed briefly. The mixture was incubated on ice for 30 minutes and then centrifuged at 11,000×g for 10 minutes at 4°C. The supernatant was completely aspirated and discarded. A final brief centrifugation step at 11,000 × g for a few seconds

was performed to remove any residual liquid. The resulting pellet contained the highly enriched lysosomal fraction.

Macrophage-Specific Listerin Adenovirus Production

The macrophage-targeted adenoviral vector was constructed by cloning R160-WT into pADM-lyz2-FH (containing the Lyz2 promoter) through restriction digestion and ligation, followed by sequence verification. HEK293A cells (0.3-0.5 × 10^6 cells/well) were transfected with PEI/DNA complexes containing 1 μg shuttle plasmid and 2 μg adenoviral backbone plasmid (AD5F35) in serum-free DMEM. Primary virus (P0) was harvested upon cytopathic effect (CPE) development and amplified in 10-cm dishes. Secondary amplification (P1) utilized P0 stock to infect fourteen 10-cm dishes of HEK293A cells. Viruses were harvested at >80% CPE, with supernatant and cell pellets processed separately. PEG-precipitated supernatants and sonicated cell pellets (30% amplitude, 30-s pulses × 4) were combined and purified via discontinuous iodixanol gradient ultracentrifugation (15%/25%/40%/60%) at 100,000 × g for 2.5 hr (4°C). The viral band was extracted, buffer-exchanged into PBS/0.001% PF68, and concentrated using 100-kDa MWCO centrifugal filters. Concentrated virus was sterile-filtered (0.22 μm), stabilized in A195 buffer, aliquoted, and stored at -80°C. Viral titers were determined by $TCID_{50}$ assay. Adenovirus was administered via tail vein injection at a dose of $3×10^9$ plaque-forming units (PFU) per mouse, with injections repeated once every two weeks. The adenovirus was administered via tail vein injection at a dose of $5×10^9$ plaque-forming units (PFU) per mouse every two weeks to achieve sustained overexpression in vivo.

ABCA1 and ABCA1-K1884/K1957 Adenovirus Construction

Recombinant adenoviruses expressing wild-type ABCA1 or lysine-mutant ABCA1-K1884/K1957 were generated by subcloning the respective sequences into the pADM-CMV-FH shuttle vector using restriction enzyme-based cloning and sequence verification. Viral production, amplification, purification, titration and administration followed identical protocols as described for the Listerin adenovirus.

AAV-shABCA1 Production

ABCA1-targeting shRNA sequences were cloned into the Pav-lyz2-miR30-GFP vector via restriction digestion/ligation and sequence verification. For AAV production, HEK293T cells (85–90% confluency) were transfected in serum-free DMEM supplemented with 1% HEPES and 1% penicillin-streptomycin using a plasmid mixture (helper:packaging:vector = 120:76:66 mass ratio) complexed with transfection reagent (786 μg). Following 72-hr incubation (37°C/5% CO_2), cells and media were harvested. Supernatants were precipitated with 8% PEG8000 (2 hr, 4°C), while cell pellets were resuspended in PBS/0.001% PF68, subjected to freeze-thaw cycling, and treated with 5 M NaCl. Combined lysates were sonicated to reduce viscosity and clarified by centrifugation (3,500 × g, 30 min, 4°C). Purification was performed using iodixanol density gradient ultracentrifugation (480,000×g, 2.5 hr, 4°C). The viral fraction (25–40% interface) was collected, concentrated to 100 μL using 100-kDa centrifugal filters, and formulated in PBS/0.001% PF68. Finally，All viral preparations underwent genomic titer quantification (qPCR), functional validation (in vitro transduction), purity

assessment (silver staining; endotoxin <0.5 EU/mL), and identity confirmation (restriction digest or sequencing).

Plasmid transfection and RNA interference

cDNA of Listerin was amplified from macrophages by standard PCR, cloned in pCMV2-Flag, GFP-tagged ABCA1 were obtained from GeneCopoeia (USA), truncated mutants were constructed by KOD-Plus-Mutagenesis Kit (TOYOBO Life Science, JAPAN), based on wild type plasmids. All constructs were verified by DNA sequencing. For the transient transfection of plasmids and siRNA duplexes, plasmids were transfected into HEK293T cells with Lipofectamine 3000 reagents (Invitrogen, USA), and plasmids were transfected into RAW294.7 with jetOPTIMUS® (Polyplus transfection, French), siRNA duplexes were transfected into macrophages with RNAimax reagents (Invitrogen, USA). The target sequences of siRNAs are as follows: mouse Listerin sequences: sense 5′-GAGCAGUUGAUCCCAUUUATT-3′, antisense 5′-UAAAUGGGAUCAACUGCUCTT-3′; mouse HRS sequences: sense 5′-GCCGUACAAUAUGCAGAAUTT-3′, antisense 5′-AUUCUGCAUAUUGUACGGCTT-3′; negative control (NC) sequences: sense 5′-UUCUCCGAACGUGUCACFUTT-3′. mouse MEMF sequences: sense 5′-UGAGAACAGUGAUGCUGAA-3′, antisense5′- ACUCUUGUCACUACGACUU-3′.

Analysis of Western blot

Total protein was extracted from mouse aortic tissue by using the Total Protein Extraction Kit for Blood Vessels (SA-03-BV, Invent Biotechnologies, Plymouth, MN, USA), and total protein was extracted from macrophages by using Cell Lysis (Sigma-

Aldrich, USA) containing protease inhibitor cocktail. Protein concentrations of extracts were measured with a BCA kit (ThermoFisher Scientific, USA), whole-cell lysates were separated by SDS–PAGE, transferred onto PVDF membranes, and then blotted with 5% BSA (Albumin from bovine serum) and incubated with indicated antibodies overnight at 4°C.

RNA quantification

Total RNA was extracted from primary peritoneal macrophages using RNeasy mini kit (Qiagen, 74106, Germany), and reverse transcription was performed using PrimeScript RT reagent kit with gDNA Eraser (TaKaRa, Japan) following manufacturer's instructions. The reverse-transcription products were amplified with the SYBR Green PCR Master Mix (Roche, Switzerland). PCR amplification conditions are as follows:95˚C at 10 min for initial denaturation, 40 cycles for amplification (95˚C at 15s for denaturation, 55˚C at the 15s for annealing, and 72˚C at the 20s for extension). The mRNA levels were normalized by β-actin expression and the 2−ΔΔCt method was used for comparison. The primer sequences are listed in Supplementary Table S2.

TUBE pulldowns

Macrophages were lysed with 1 mL Cell Lysis buffer (50mM Tris-HCl, pH 7.5, 0.15M NaCl, 1mM EDTA, 1% NP-40, 10% glycerol.) containing 1x protease inhibitors cocktail. The cell lysate was centrifuged at 14,000g for 10min to remove cell debris and other insoluble proteins. The supernatant was retained and an "INPUT" sample was taken from it for western blotting analysis, then add 30μL equilibrated Magnetic-TUBE (TUBE1-Magnetic Beads, LifeSensors, USA) to remaining clarified cell lysate and

incubate 2 hours at 4°C on a rocker platform. Place the tube into a magnetic stand and collect the beads, then wash the beads with 1ml TBST four times, eluted with 2x SDS-PAGE Sample Buffer. The input and eluate of endogenous ubiquitin were analyzed by immunoblotting. Not surprisingly, we found that 30μL beads were sufficient to precipitate ubiquitinated proteins in the supernatant.

Extract cell surface proteins

For analysis of cell surface proteins, biotinylation of cell surface proteins was carried out as previously described. After macrophages were incubated with 0.5 mg/mL sulfo-NHS-SS-biotin (ThermoFisher Scientific, USA) for 30 minutes at 4°C with shaking. Washed the cells twice with cold PBS and quenched the reaction with 50mM glycine in PBS, then cells were lysed with Cell Lysis (Sigma-Aldrich, USA) containing protease inhibitor cocktail. The supernatant was collected after centrifugation at 12,000 g for 10 minutes and protein concentrations were measured and adjusted by a BCA kit (ThermoFisher Scientific, USA). Then, 100 μg of supernatant was incubated with 100ul of Pierce NeutrAvidin agarose beads (ThermoFisher Scientific, USA) for at least 12 hours at 4°C, the beads were washed three times with PBS containing a protease inhibitor cocktail. Finally eluted with 2x SDS-PAGE Sample Buffer, boiled at 100°C for five mins, and analyzed membrane and total proteins by immunoblotting.

Immunoprecipitation (IP) and Ubiquitination assay

Macrophages and HEK293T cells were lysed with 1 ml IP buffer (150 mM saline, 50 mM Tris–HCl, 1% NP-40, PH7.8) containing 1x protease inhibitors cocktail (Merck, Darmstadt, Germany), the supernatant was collected after centrifugation at 13000g for

10min and concentrations between the different samples were adjusted by the BCA assay (ThermoFisher Scientific, USA), behind the supernatant was incubated with protein A/G Plus–Agarose immunoprecipitation reagent (Santa Cruz Biotechnology, USA) for 1 hour, added 1μg corresponding antibody and incubate overnight at 4°, then washed beads with IP buffer for four times and eluted with 2x SDS-PAGE Sample Buffer, boiled at 100°C for five mins and analyzed by immunoblotting.

To the analysis of the polyubiquitination of ABCA1 in HEK293Tcells. GFP–ABCA1, HA-ubiquitin (WT), HA–ubiquitin (K48), HA-ubiquitin(K63), and Flag-Listerin (WT) or its mutant plasmids were transfected into HEK293Tcells, then whole-cell extracts were immunoprecipitated with the GFP-specific antibody and analyzed by immunoblot with anti-HA. For the analysis of the polyubiquitination of ABCA1 in primary peritoneal macrophages, the cells were incubated with low oxidative density (ox-LDL) the cells, furthermore, the immunoprecipitation of whole cell extracts was performed with ubiquitin, ubiquitin (K48) and ubiquitin(K63) antibody.

Immunofluorescence staining

For tissue staining, paraffin sections of human carotid artery samples were deparaffinized and hydrated, and then antigen retrieval with Citrate Antigen Retrieval solution (CWBIO, China) for 10 minutes before blocking and staining, cryosections of aortic root tissue was hydrated for 10 minutes, then fixed with ice-cold acetone for 10 minutes. slides blocked with 5% goat serum for 1 hour at room temperature. Furthermore, sections were incubated with primary antibodies overnight at 4°C, next day they were respectively incubated with fluorescently labeled secondary antibodies

(Alexa Flour 594, 488;1:200) for 1 hour at room temperature. Slides were washed with PBS three times and stained with Mounting Medium with DAPI (Abcam, UK).

For cellular immunofluorescence, primary peritoneal macrophages were seeded in a 12-well plate with a cover glass. After incubation with ox-LDL, cells were washed in PBS and fixed with 1% paraformaldehyde for 15 min at room temperature, the cells were blocked with 5% goat serum in PBS at room temperature for 1 hour and incubated with the relevant primary antibodies in PBS overnight at 4℃, next day they were respectively incubated with fluorescently labeled secondary antibodies (Alexa Flour 594, 488;1:200) for 1 hour at room temperature. Slides were washed with PBS three times and stained with Mounting Medium with DAPI (Abcam, UK). Sections reacting with non-immune IgG and secondary antibodies served as negative controls. Finally, we used an electric upright microscope (DS-Ri2, Nikon, Japan) for the image.

LC-MS/MS analysis of ABCA1 ubiquitination site

To detect the protein ubiquitination sites of ABCA1, GFP-ABCA1 co-transfected with Flag-Listerin and HA-ubiquitin, or GFP-ABCA1 only with HA-ubiquitin into HEK293T cells for 24 hours. Following, the cell lysates were immunoprecipitated with anti-GFP affinity beads, and beads were washed with IP buffer three times, then boiled and separated by SDS–PAGE. After Coomassie Blue staining, ABCA1-specific bands were cut and analyzed by tandem mass spectrometry (PTM Biolabs, China). The resulting MS/MS data were processed by Proteome Discoverer 1.3.

Supplementary Figures

FigureS1

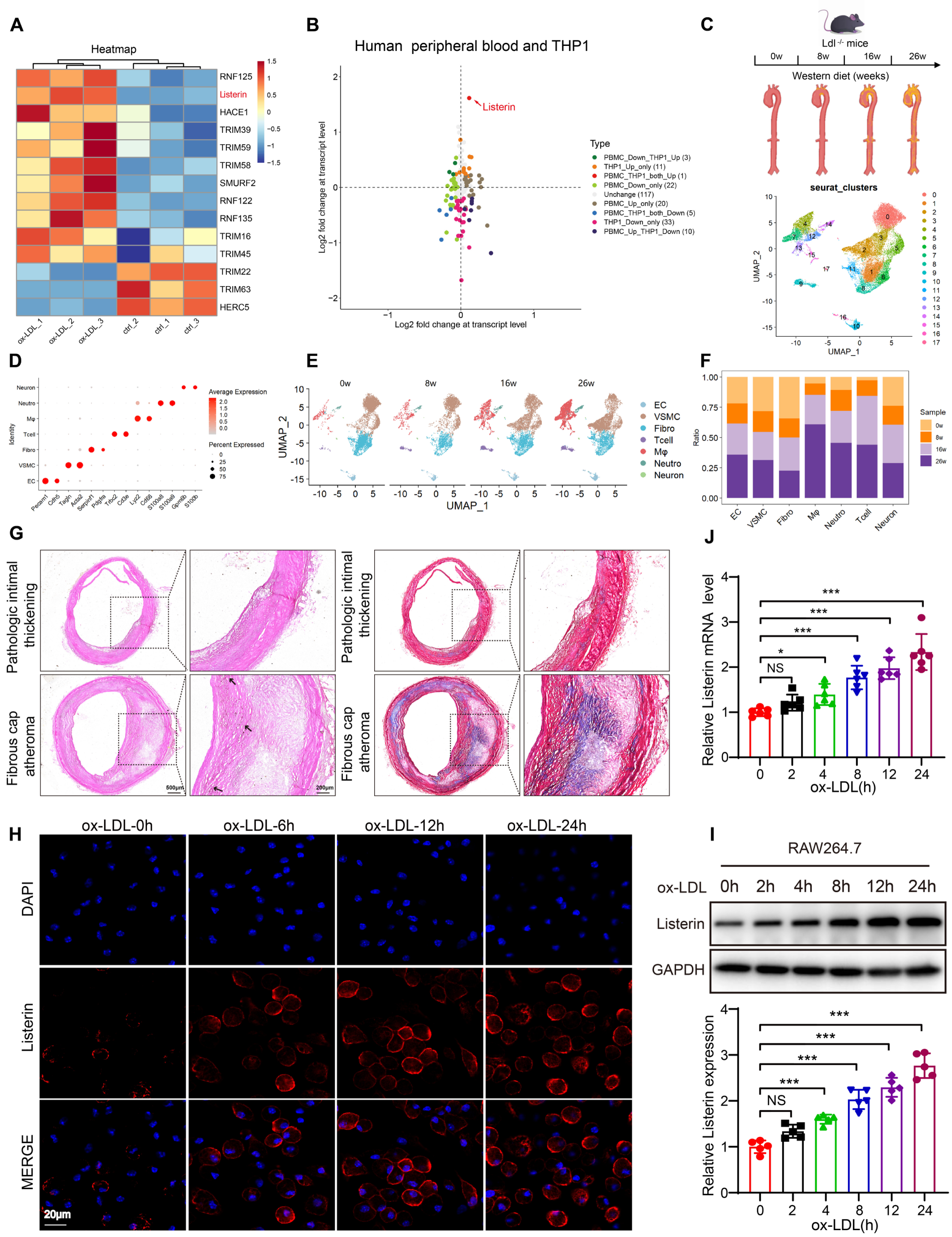

FigureS1. Listerin expression increases in human and mouse atherosclerotic plaque tissues **(A)** Heatmap illustrating RNA sequencing data of E3 ubiquitin ligase from THP1 cells incubation with ox-LDL for 6h. **(B)** Quadrantal diagram of RNA sequencing

of THP1 cells after ox-LDL stimulation and microarrays of PBMC from human with or without carotid artery atherosclerosis (GSE23746). **(C)** Schematic of scRNA-seq of Ldlr-/- mice fed with western diet (WD) for various time points (0, 8, 16, 26 weeks). Unbiased clustering of cells from all samples revealed 18 clusters. **(D)** Dotplot overview of expression of key marker genes identified for the seven cell types. **(E)** UMAP plot was used to directly visualize the variations in the composition and number of cell types in mouse atherosclerotic plaques across different time points. **(F)** Bar plot showing the changes in the proportions of the cell types among four timepoints (0, 8, 16, 26 weeks) of atherosclerotic plaques. **(G)** Von Kossa and Masson's Trichrome staining of human coronary atheroma to determine the calcification and fibrosis of plaque. Scale bar=500μm, 200μm. **(H)** Confocal microscopic images of Listerin expression in mouse peritoneal macrophages (PMs) were incubated with oxLDL (50μg/mL) at different times. n = 5 per group. Scale bar=20μm. **(I)** Immunoblot analysis of Listerin expression in RAW264.7 macrophages after oxLDL (50μg/mL) treatment. n = 5 per group. **(J)** Quantitative RT-PCR analysis of Listerin mRNA levels in RAW264.7 macrophages after ox-LDL (50μg/mL) treatment at the indicated time. n = 6 per group. Data were presented as mean ± SD and the Shapiro–Wilk method was used to test the normal distributions. Statistical analysis was performed by One-way ANOVA with Dunnett post hoc test. For multiple-group comparisons, the adjusted P-values are provided, NS (not significant) $P > 0.05$, $^*P < 0.05$, $^{***}P < 0.001$. Each experiment was obtained at least three times independently.

FigureS2

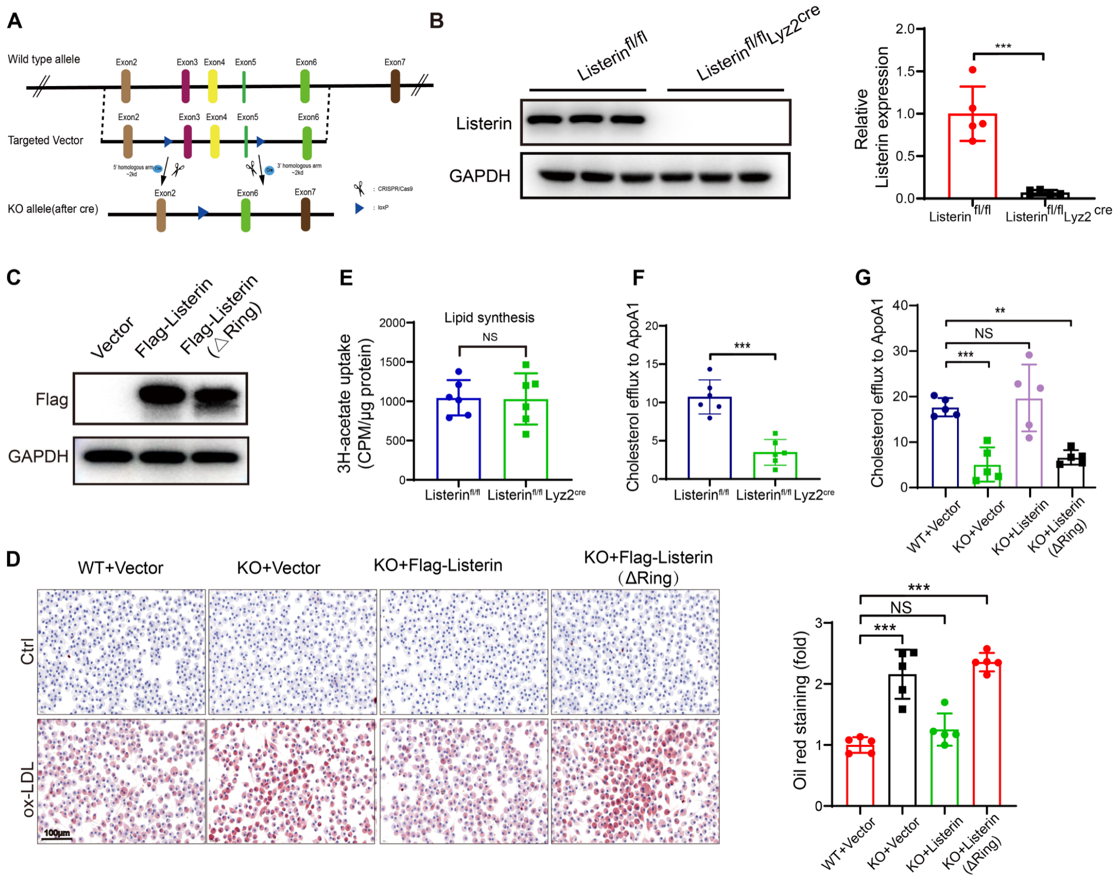

FigureS2. Macrophage Listerin deficiency inhibits cholesterol efflux and aggravates foam cell formation **(A)** Schematic diagram to generate Listerin$^{fl/fl}$Lyz2^{cre} mice. **(B)** Immunoblot analysis of Listerin expression in peritoneal macrophages (PMs) from Listerin$^{fl/fl}$ and Listerin$^{fl/fl}$Lyz2^{cre} mice. n=5 per group. **(C)** Immunoblot analysis of Flag–Listerin and Flag–Listerin-ΔRing expression in PMs after adenovirus-mediated overexpression of Flag-Listerin and Flag–Listerin-ΔRing for 24 h. **(D)** Oil red O staining images and quantitation in Listerin$^{fl/fl}$ and Listerin$^{fl/fl}$Lyz2^{cre} PMs incubated with or without oxLDL (50 μg/mL) for 24 hours after infecting with indicated adenovirus. n=5 per group. Scale bar=100μm. **(E)** PMs isolated from Listerin$^{fl/fl}$ and Listerin$^{fl/fl}$Lyz2^{cre} mice were first incubated with oxLDL. Subsequently, the cells were stimulated with 4 μCi/mL of Acetic acid [3^{H}] for 4 hours. Lipids were then extracted

and subjected to liquid scintillation counting to measure lipid synthesis activity. n=6 per group. **(F)** ApoA1-mediated cholesterol efflux assay of PMs isolated from $Listerin^{fl/fl}$ and $Listerin^{fl/fl}Lyz2^{cre}$ mice. n=6 per group. **(G)** ApoA1-mediated cholesterol efflux assay of PMs isolated from $Listerin^{fl/fl}$ and $Listerin^{fl/fl}Lyz2^{cre}$ mice and then infected with indicated adenovirus. n=5 per group. Data were presented as mean ± SD and the Shapiro–Wilk method was used to test the normal distributions. Statistical analysis was performed by Student's *t*-test for B, E and F. One-way ANOVA with Dunnett post hoc test was used for D and G. NS (not significant) P > 0.05, **P < 0.01, ***P < 0.001.

FigureS3

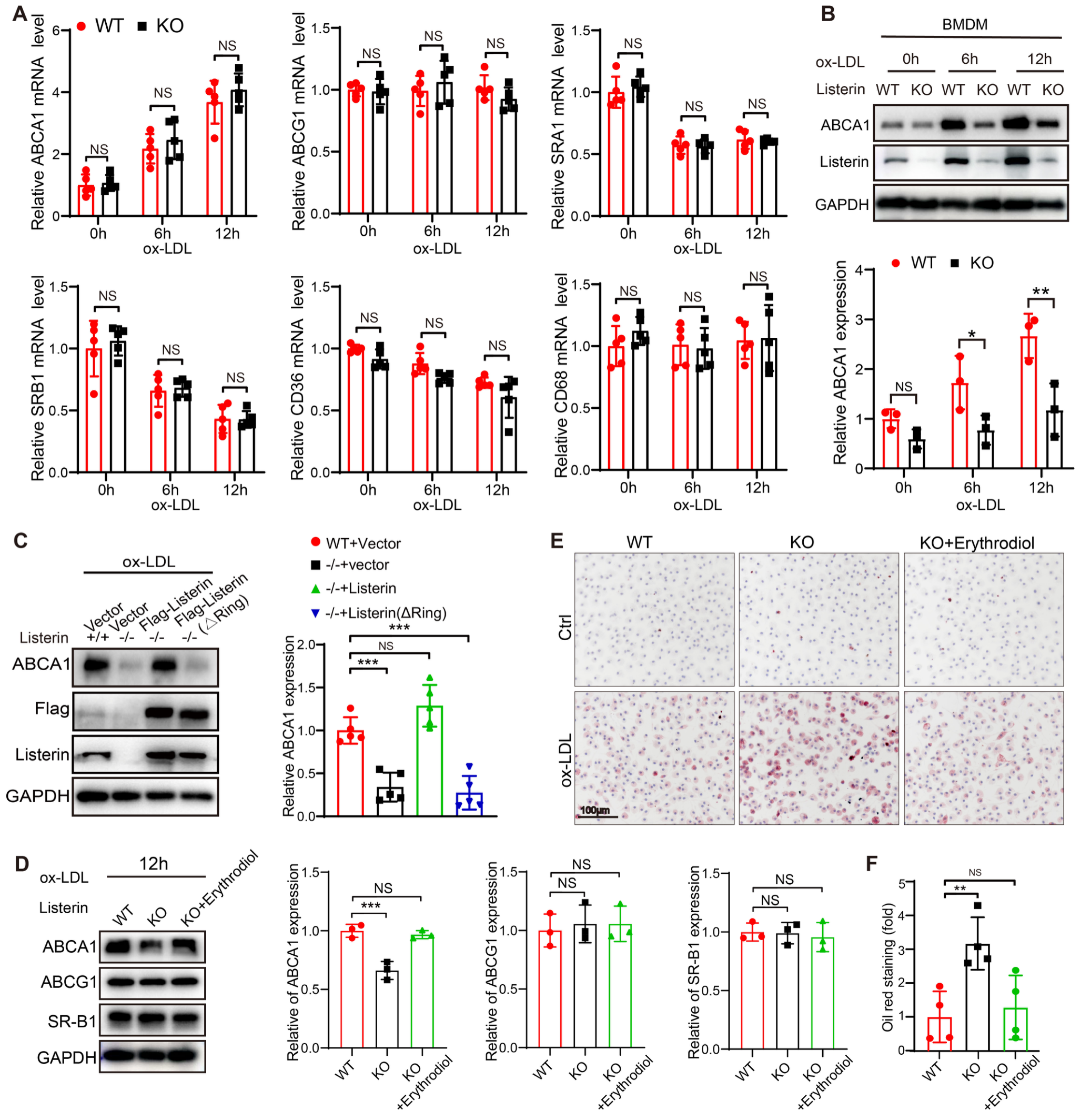

FigureS3. Listerin deficiency promotes foam cell formation through downregulating ABCA1 **(A)** Quantitative RT-PCR analysis of ABCA1, ABCG1, SR-A1, SR-B1, CD36, and CD68 mRNA levels in Listerin$^{fl/fl}$ and Listerin$^{fl/fl}$Lyz2^{cre} mice PMs after oxLDL (50 µg/mL) treatment. n=5 per group. **(B)** Immunoblot analysis and quantitation analysis (Lower panel) of ABCA1 expression in Listerin$^{fl/fl}$ and Listerin$^{fl/fl}$Lyz2^{cre} BMDMs after oxLDL incubation. n=3 per group. **(C)** Immunoblot analysis and quantitation analysis of ABCA1 expression in Listerin$^{fl/fl}$ and Listerin$^{fl/fl}$Lyz2^{cre} PMs infected with indicated

adenovirus. n=5 per group. **(D)** Immunoblot analysis of ABCA1, ABCG1, and SR-B1 expression in $Listerin^{fl/fl}$ and $Listerin^{fl/fl}Lyz2^{cre}$ PMs pre-incubated with ABCA1 agonist Erythrodiol or DMSO, and then incubated with or without oxLDL (50 μg/mL) for 24 hours. n=3 per group. **(E)** Oil red O staining images and quantitation **(F)** in $Listerin^{fl/fl}$ and $Listerin^{fl/fl}Lyz2^{cre}$ PMs. PMs pre-incubated with or without ABCA1 agonist Erythrodiol, followed by incubation with or without oxLDL (50 μg/mL) for 24 hours. n=4 per group. Scale bar=100μm. Data were presented as mean ± SD and the Shapiro–Wilk method was used to test the normal distributions. Statistical analysis was performed with Two-way ANOVA followed by Sidak post hoc test for A and B. One-way ANOVA with Dunnett post hoc test for C, D and F. For multiple-group comparisons, the adjusted P-values are provided, NS (not significant) $P > 0.05$, $^*P < 0.05$, $^{**}P < 0.01$ and $^{***}P < 0.001$.

FigureS4

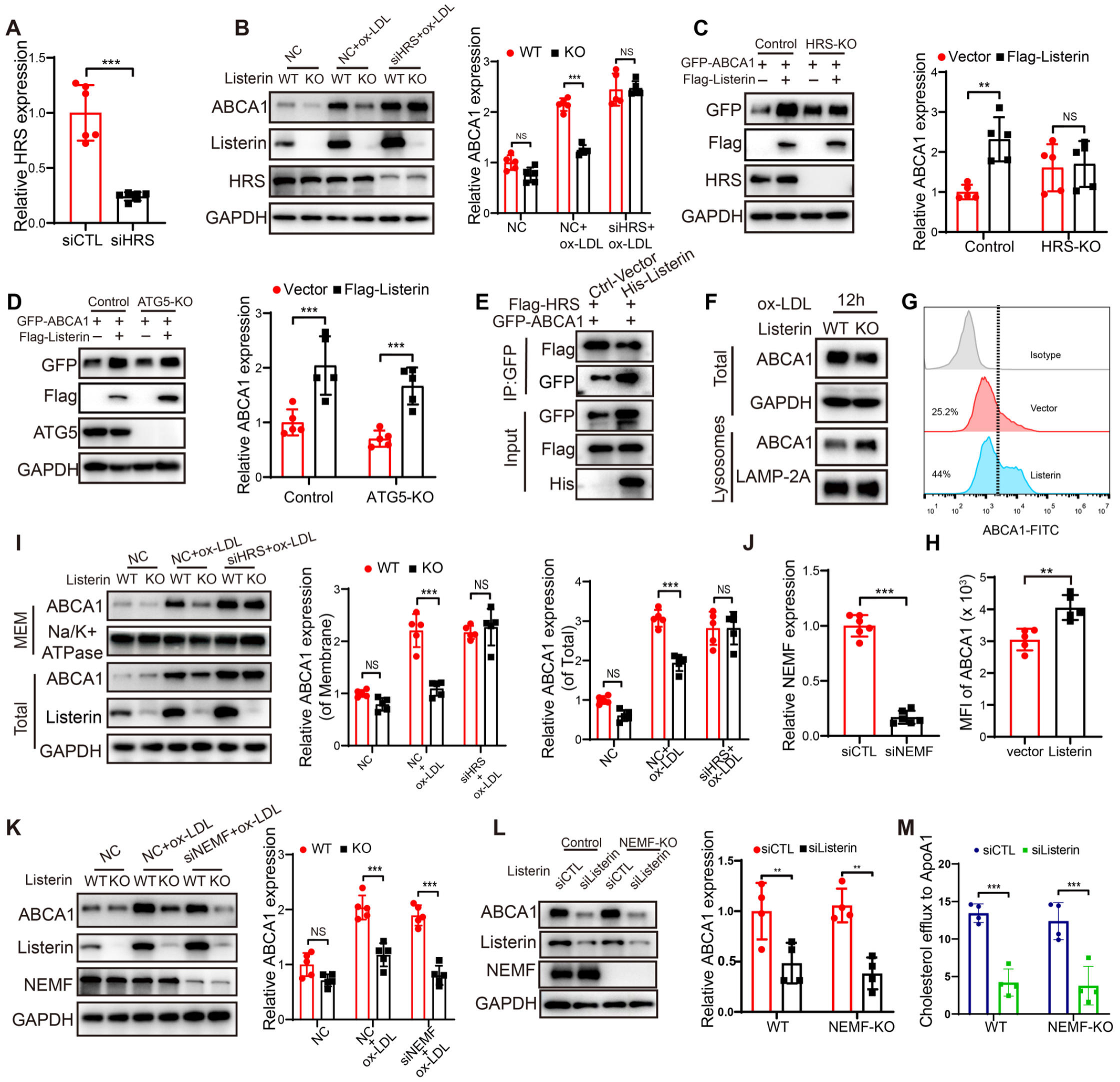

FigureS4. Listerin inhibits the degradation of ABCA1 through the ESCRT-lysosomal pathway **(A)** Quantification of HRS expression. n=6 per group. **(B)** Immunoblot and quantitative analysis of ABCA1 expression in PMs from Listerin$^{fl/fl}$ and Listerin$^{fl/fl}$ Lyz2^{cre} mice with HRS silencing followed by oxLDL stimulation (50 µg/mL). n=5 per group. **(C-D)** Immunoblot analysis of GFP-ABCA1 expression in HRS KO or ATG5 KO HEK293T cells. n=5 per group. **(E)** Co-IP assay of Flag-HRS with GFP-ABCA1 with or without His-Listerin overexpression in HEK293T cells. **(F)** The amount of ABCA1 in whole cell lysate and lysosome. **(G-H)** Flow cytometry analysis of membrane ABCA1 in PMs after adenovirus-mediated overexpression of Flag-Listerin**.**

n=5 per group. **(I)** Immunoblot analysis of membrane (MEM) and total ABCA1 in PMs from Listerin$^{fl/fl}$ and Listerin$^{fl/fl}$Lyz2^{cre} mice with HRS silencing followed by oxLDL stimulation (50 μg/mL). n=5 per group. **(J)** Quantification of NEMF expression. n=6 per group. **(K)** Immunoblot analysis ABCA1 expression in PMs from Listerin$^{fl/fl}$ and Listerin$^{fl/fl}$Lyz2^{cre} mice with NEMF silencing followed by oxLDL stimulation (50 μg/mL). n=5 per group. **(L)** Immunoblot analysis of ABCA1 expression in NEMF KO THP-1 cells. n=4 per group. **(M)** ApoA1-mediated cholesterol efflux assay of WT and NEMF-KO THP-1 macrophages transfected with siCTL and siListerin. n=4 per group. Data were presented as mean ± SD and the Shapiro–Wilk method was used to test the normal distributions. Data analysis was performed with Student's *t*-test for H, J and Mann-Whitney Test for A, the others with Two-way ANOVA followed by Sidak post hoc test. For multiple-group comparisons, the adjusted P-values are provided, NS (not significant) P > 0.05, **P < 0.01, and ***P < 0.001. Each experiment was repeated at least three times independently.

Figure S5

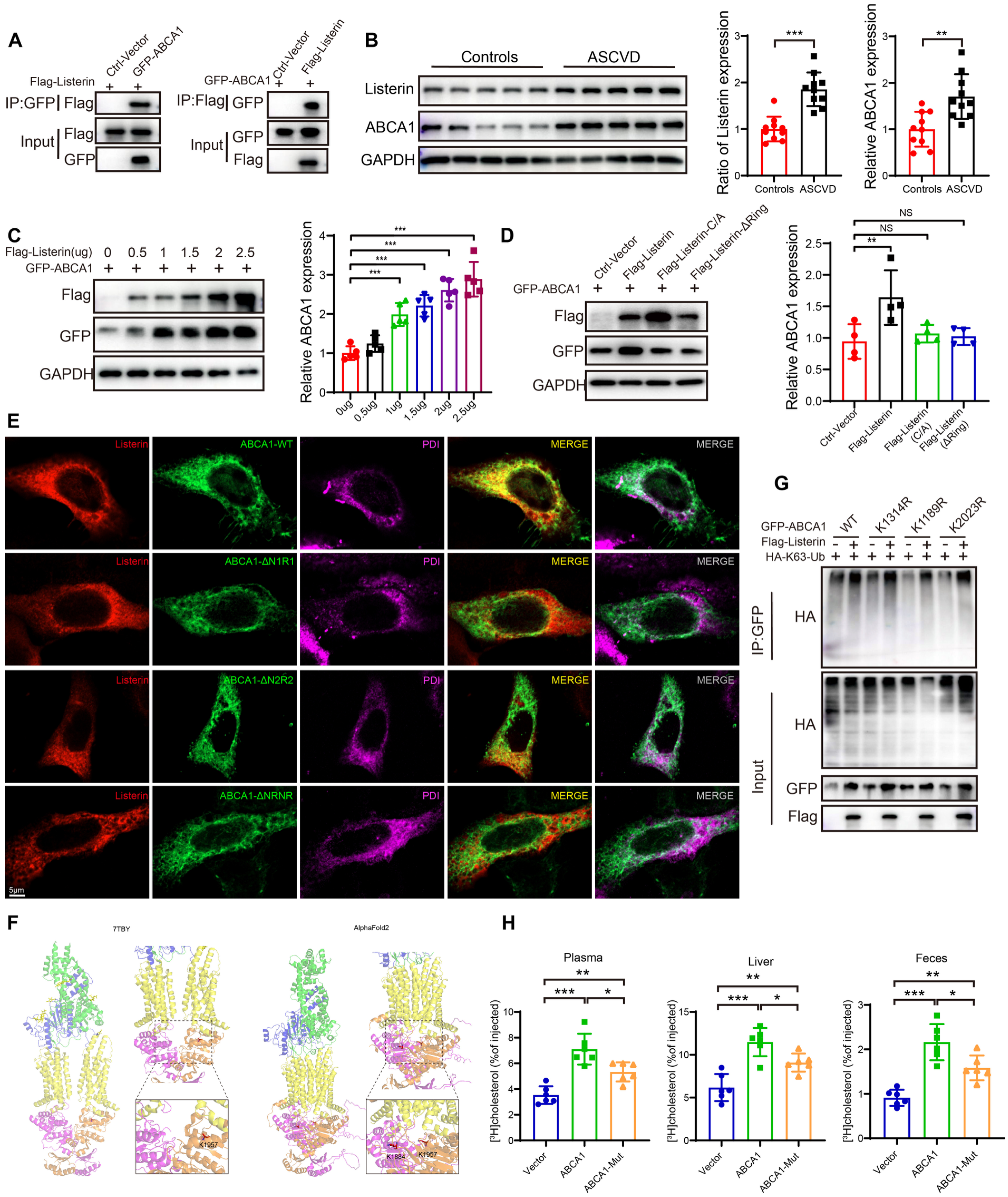

FigureS5. Mutation of the ubiquitylation sites Lys1884 and Lys1957 of ABCA1 inhibit cholesterol efflux **(A)** Co-IP assay of Flag-Listerin with GFP-ABCA1 in HEK293T cells. **(B)** Immunoblot analysis of Listerin and ABCA1 expression in peripheral blood mononuclear cells (PBMC) from Controls and ASCVD patients. n=10 per group. **(C)** Immunoblot analysis of GFP-ABCA co-transfected with an elevated concentration gradient of Flag-Listerin into HEK293T cells. n=5 per group. (**D)**

Immunoblot images of GFP-ABCA1 co-transfected with Flag-Listerin, Flag-Listerin-ΔR, and Flag-Listerin-C/A in HEK293T cells. n=5 per group. **(E)** Confocal microscopic images for Listerin, ABCA1 (WT and mutants) and endoplasmic reticulum (ER) in Hela cells. Scale bar=5μm. **(F)** The diagram of structural localization of ubiquitination sites K1884 and K1957 in human ABCA1. **(G)** Co-IP analysis of the polyubiquitination of ABCA1(WT) and its mutants in HEK293T cells transfected with GFP-ABCA1 (WT or mutants), Flag-Listerin, and HA-ubiquitin (K63). **(H)** The percent appearance of [3^{H}] cholesterol in plasma, liver, and feces 48 h after transplanting cholesterol-loaded RAW 264.7 macrophages transfected with Vector, ABCA1 or ABCA1-K1884/K1957 plasmids, n=6 per group. Data were presented as mean ± SD and the Shapiro–Wilk method was used to test the normal distributions. Student's *t*-test for B. One-way ANOVA with Dunnett post hoc test for C and D. One-way ANOVA with Tukey post hoc test for H. For multiple-group comparisons, the adjusted P-values are provided, NS (not significant) $P > 0.05$ and ***$P < 0.001$. Each experiment was repeated at least three times independently.

FigureS6

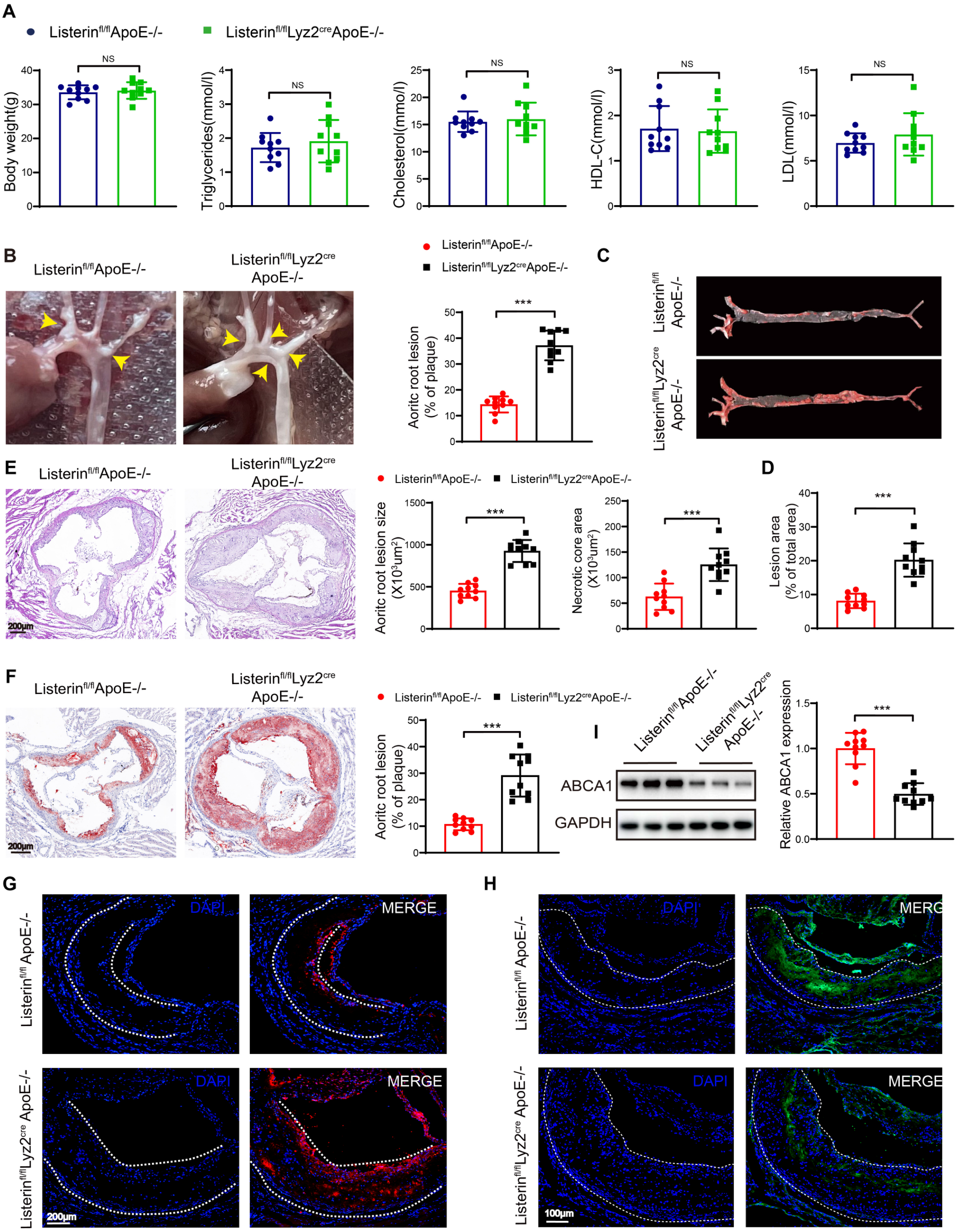

FigureS6. Listerin knockout aggravates the development of atherosclerosis in vivo

Female ApoE-/- Listerin$^{fl/fl}$ and ApoE-/- Listerin$^{fl/fl}$ Lyz2^{cre}mice were fed a Western diet for 16 weeks. **(A)** The measurement of Body weight and serum levels of triglycerides (mmol/l), cholesterol (mmol/l), HDL-C (mmol/l), and LDL (mmol/l). n=10 per group.

(B) Representative images of aortic arch regions containing white plaques (yellow arrows). **(C)** En face Oil red O staining and quantification analysis **(D)** of atherosclerotic plaques in the whole aorta. n=10 per group. **(E)** H & E staining of representative aortic root sections, quantification of lesions area and necrotic core area. n=10 per group. Scale bar=200μm. **(F)** Oil red O stained cross sections analysis of atherosclerotic plaques in the aortic root. n=10 per group. Scale bar=200μm. Immunofluorescence staining of CD68 **(G)**, and ABCA1 **(H)** in aortic root. n=10 per group. Scale bar=100μm and 200μm. **(I)** Immunoblot images and quantitative analysis of ABCA1 in the whole-aorta lysates from ApoE-$^{/-}$ Listerin$^{fl/fl}$ and ApoE-/- Listerin$^{fl/fl}$Lyz2^{cre} mice, n=10 per group. Data were presented as mean ± SD and the Shapiro–Wilk method was used to test the normal distributions. Statistical analysis was performed by Student's *t*-test. ***P < 0.001. Each experiment was repeated at least three times independently.

FigureS7

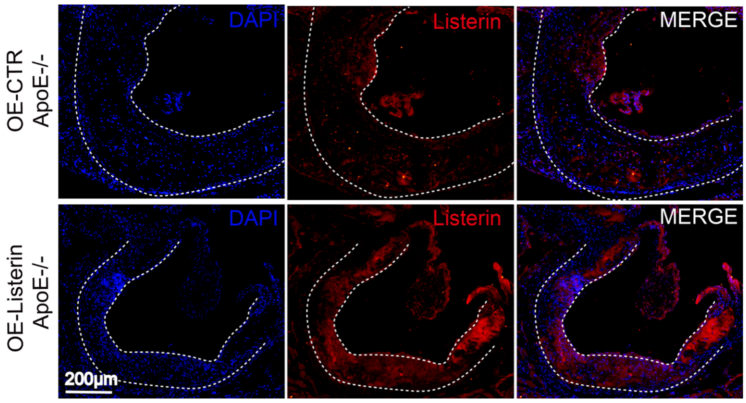

FigureS7. Listerin overexpression ameliorates the development of atherosclerosis **(A)** Immunofluorescence staining of Listerin in aortic root. n=8 per group. Scale bar=200μm. Each experiment was repeated at least three times independently.

FigureS8

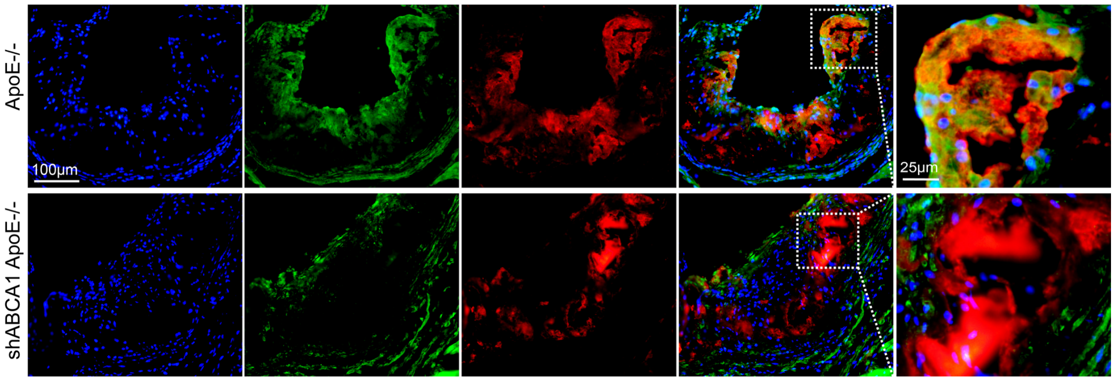

FigureS8.Listerin Overexpression Attenuates Atherosclerotic Progression via ABCA1-Dependent Regulation in vivo **(A)** Immunofluorescence staining of ABCA1 in aortic root. n=6 per group. Scale bar=100μm. Each experiment was repeated at least three times independently.

FigureS9

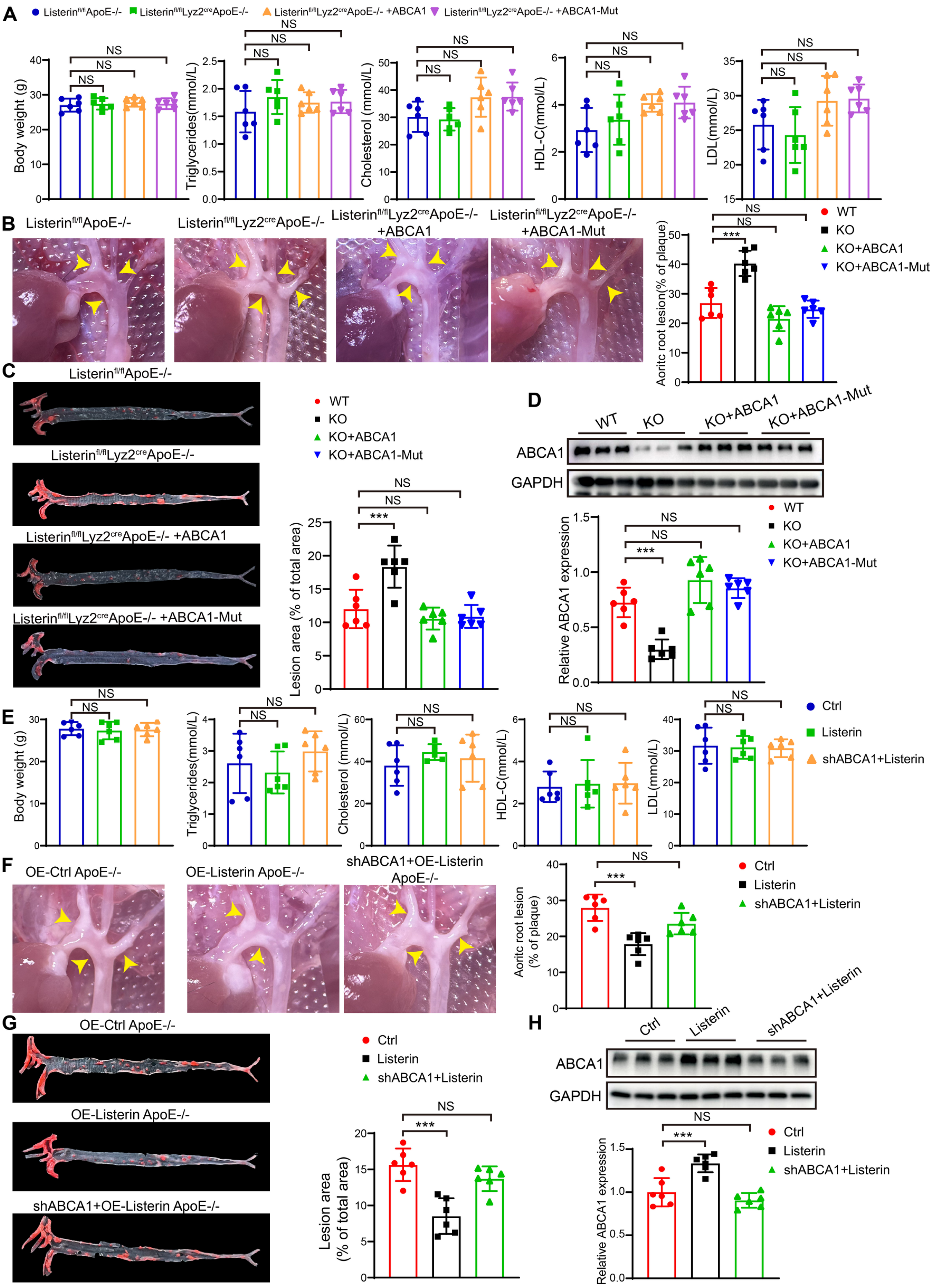

FigureS9. Listerin regulates the progression of atherosclerosis through ABCA1

(A) The measurement of Body weight and serum levels of triglycerides (mmol/l), cholesterol (mmol/l), HDL-C (mmol/l), and LDL (mmol/l). n=6 per group. **(B)**

Representative images of aortic arch regions containing white plaques (yellow arrows). **(C)** En face Oil red O staining and quantification of atherosclerotic plaques in the whole aorta. n=6 per group. **(D)** Immunoblot images and quantitative analysis of ABCA1 in the whole-aorta lysates from ApoE-/- Listerin$^{fl/fl}$, ApoE-/- Listerin$^{fl/fl}$ Lyz2^{cre}, ApoE-/- Listerin$^{fl/fl}$ Lyz2^{cre}+ABCA1 and ApoE-/- Listerin$^{fl/fl}$ Lyz2^{cre}+ABCA1-Mut mice, n=6 per group. **(E)** The measurement of Body weight and serum levels of triglycerides (mmol/l), cholesterol (mmol/l), HDL-C (mmol/l), and LDL (mmol/l). n=6 per group. **(F)** Representative images of aortic arch regions containing white plaques (yellow arrows). **(G)** En face Oil red O staining and quantification of atherosclerotic plaques in the whole aorta. n=6 per group. **(H)** Immunoblot images and quantitative analysis of ABCA1 in the whole-aorta lysates from OE-Ctrl ApoE-/-, OE-Listerin ApoE-/-, and shABCA1+OE-Listerin ApoE-/- mice, n=6 per group. Data were presented as mean ± SD and the Shapiro–Wilk method was used to test the normal distributions. One-way ANOVA followed by Dunnett post hoc test is used if the data is normally distributed, and Kruskal-Wallis followed by Dunnett post hoc test is used if not. NS (not significant) $P > 0.05$, and ***$P < 0.001$. Each experiment was repeated at least three times independently.
