# Supplementary material for: E3 ubiquitin ligase Listerin regulates macrophage cholesterol efflux and atherosclerosis by targeting ABCA1
Source: J Clin Invest. 2025 Jun 17;135(16):e186509. doi: 10.1172/JCI186509 (PMC12352907; doi:10.1172/JCI186509)

Full unedited gel for Figure 1H

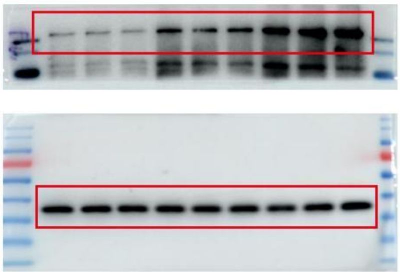

Full unedited gel for Figure 1I

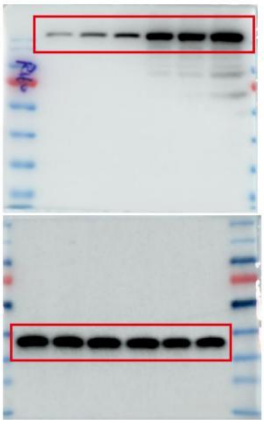

Full unedited gel for Figure 3C

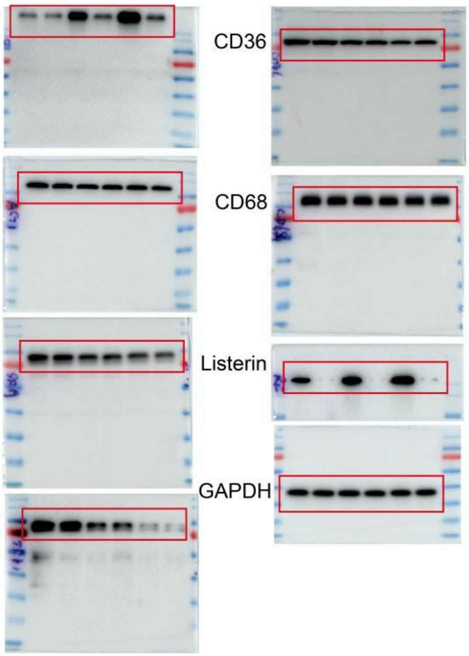

Full unedited gel for Figure 3D

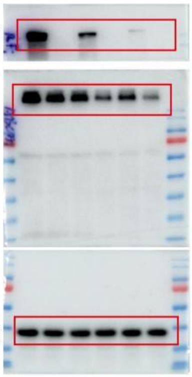

Full unedited gel for Figure 3E

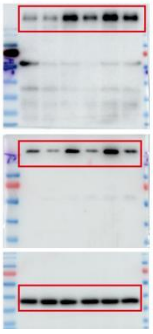

Full unedited gel for Figure 3F

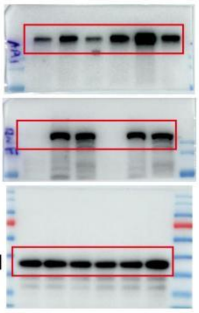

Full unedited gel for Figure 4A

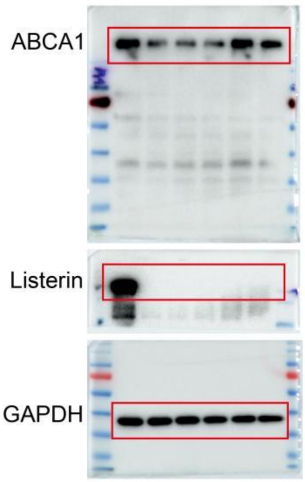

Full unedited gel for Figure 4B

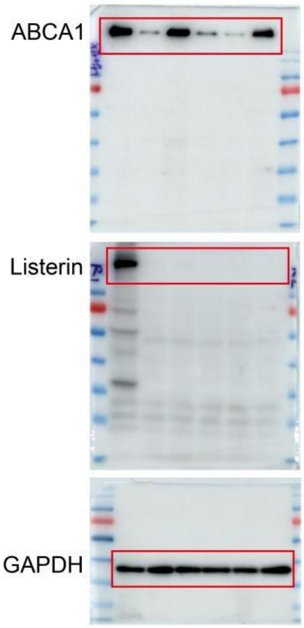

Full unedited gel for Figure4C

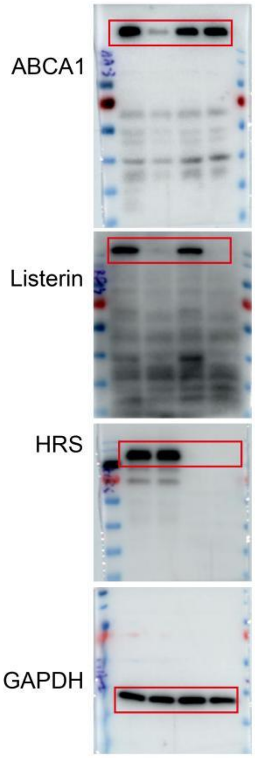

Full unedited gel for Figure4E

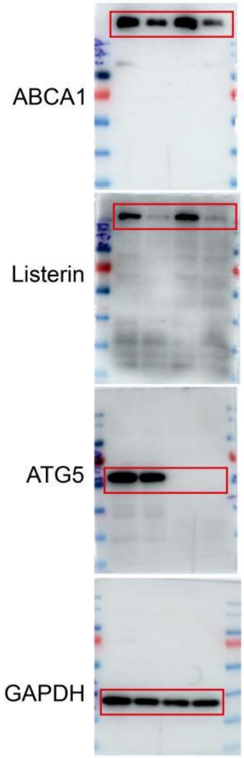

Full unedited gel for Figure 4G

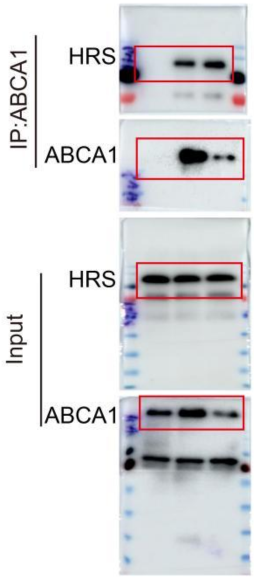

Full unedited gel for Figure 4J

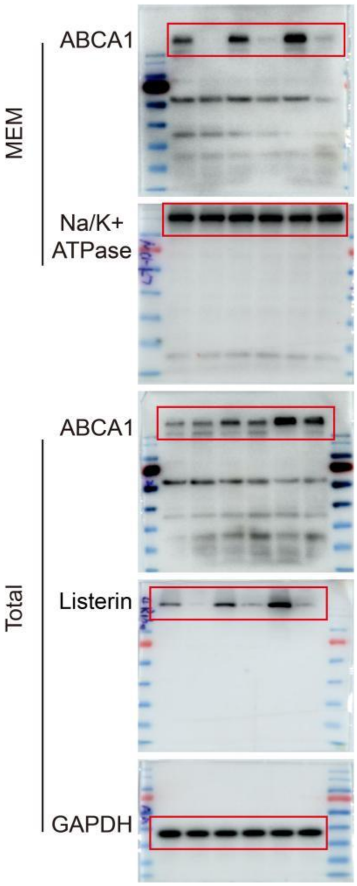

Full unedited gel for Figure 5A

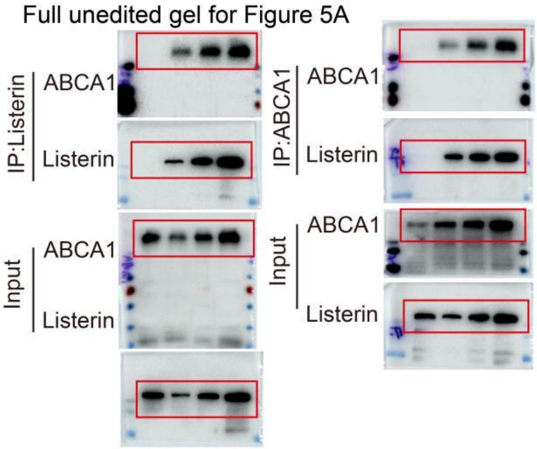

Full unedited gel for Figure 5B

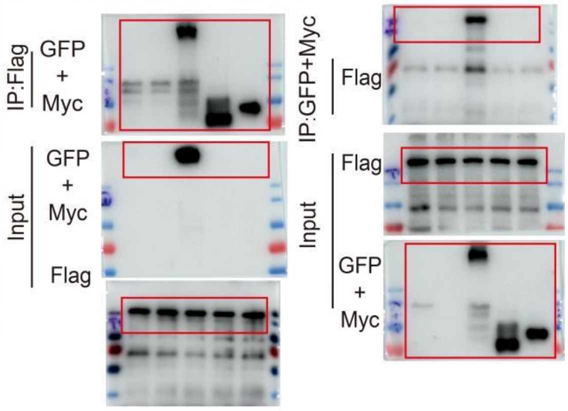

Full unedited gel for Figure 5I

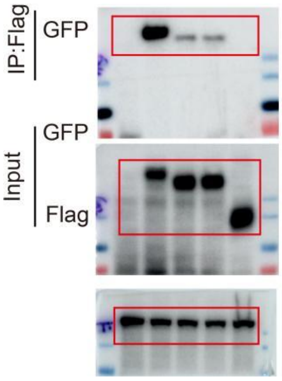

Full unedited gel for Figure 5K

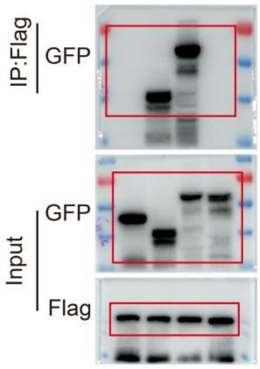

Full unedited gel for Figure 6A

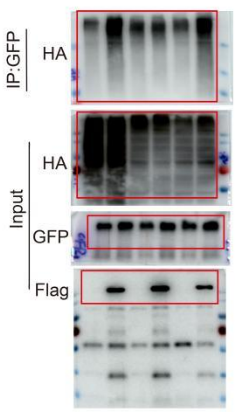

Full unedited gel for Figure 6B

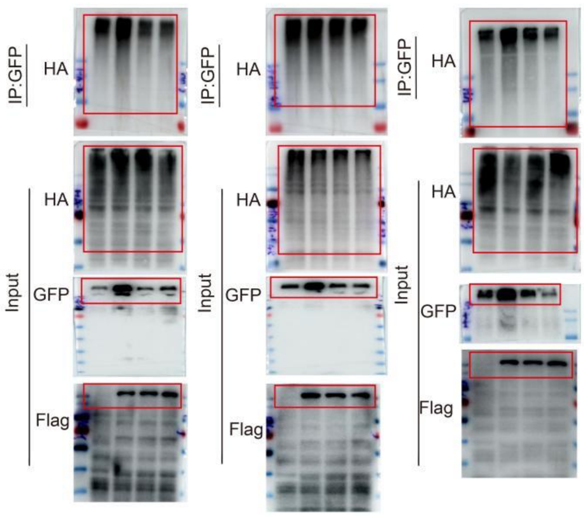

Full unedited gel for Figure 6I

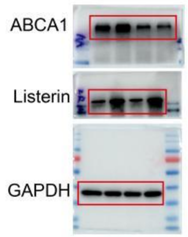

Full unedited gel for Figure 6C

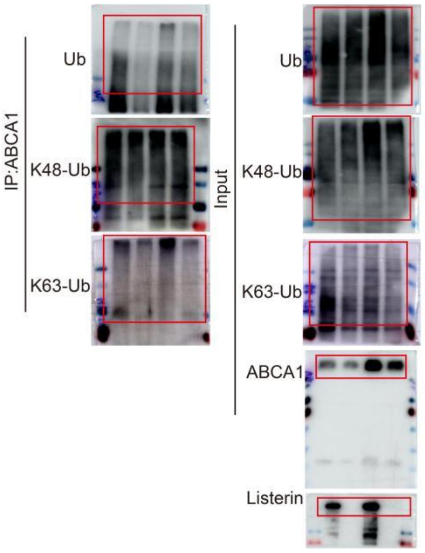

Full unedited gel for Figure 6D

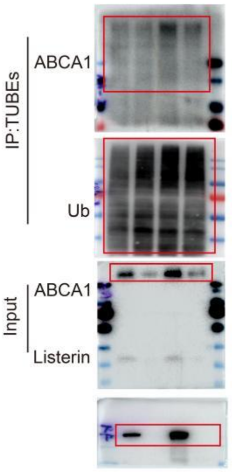

Full unedited gel for Figure 6F

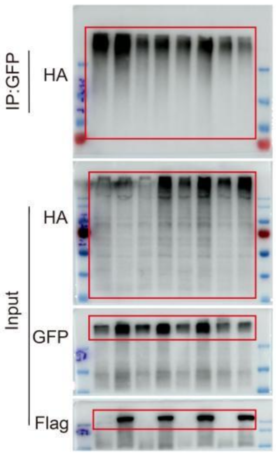

Full unedited gel for Figure 7I

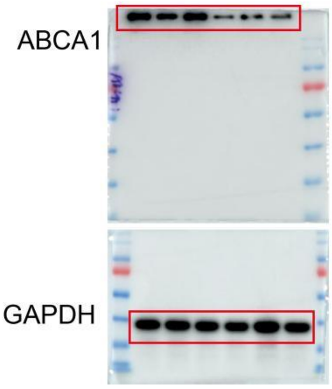

Full unedited gel for Figure 8I

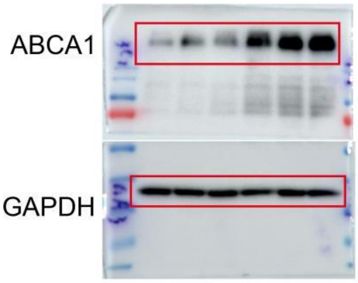

Full unedited gel for Figure S1I

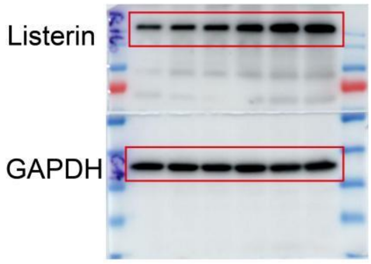

Full unedited gel for Figure S2B

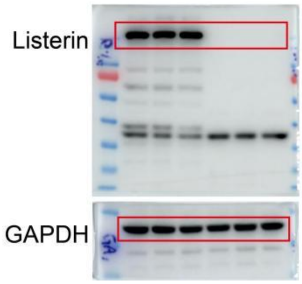

Full unedited gel for FigureS2C

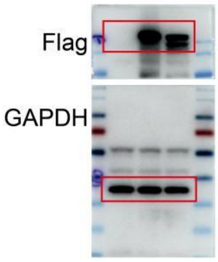

Full unedited gel for Figure S3B

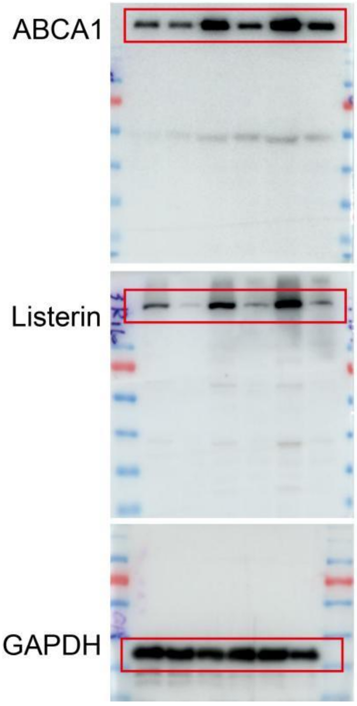

Full unedited gel for Figure S3C

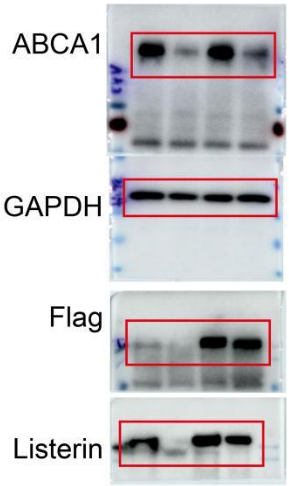

Full unedited gel for Figure S3D

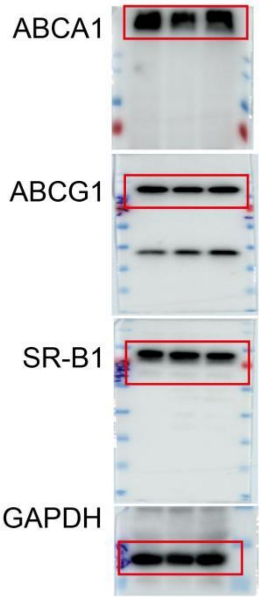

Full unedited gel for Figure S4B

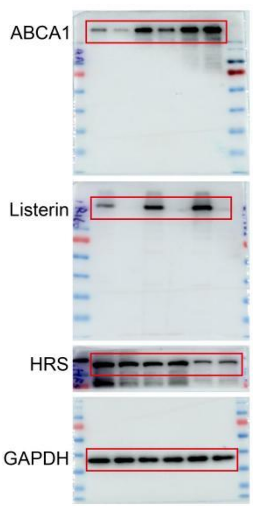

Full unedited gel for FigureS4C

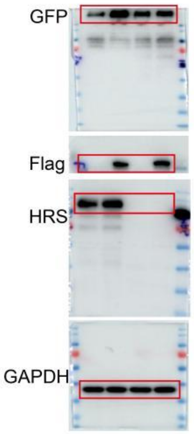

Full unedited gel for FigureS4D

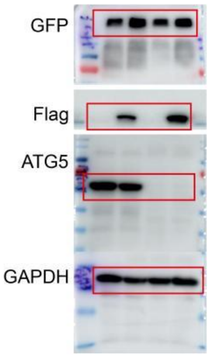

Full unedited gel for FigureS4E

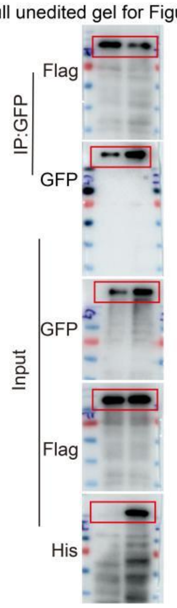

Full unedited gel for FigureS4F

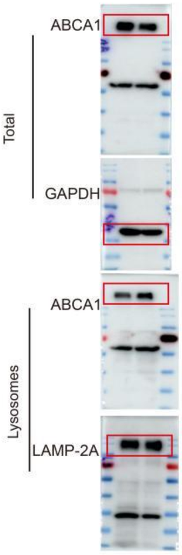

Full unedited gel for Figure S4I

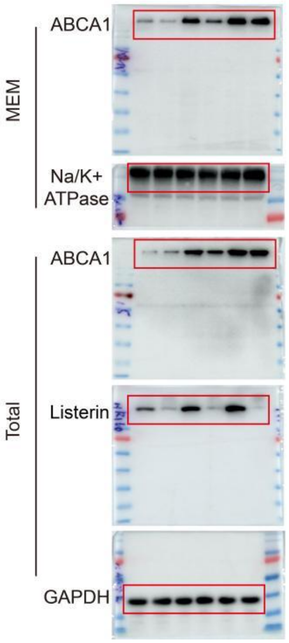

Full unedited gel for FigureS4K

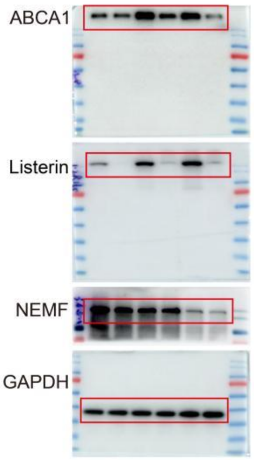

Full unedited gel for FigureS4L

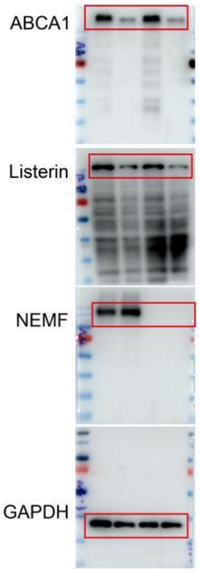

Full unedited gel for Figure S5A

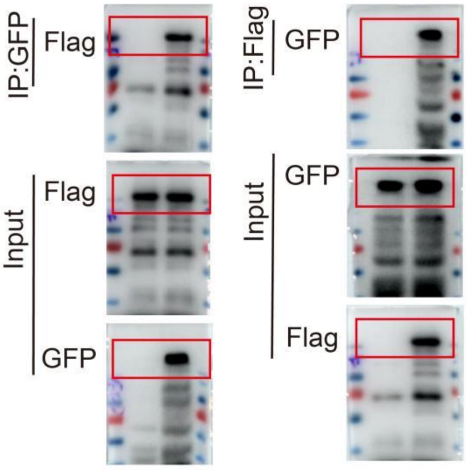

Full unedited gel for Figure S5B

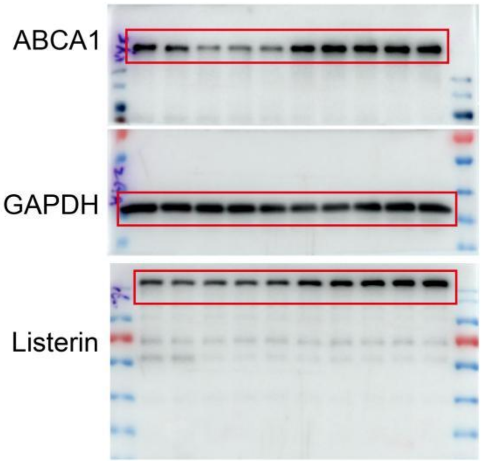

Full unedited gel for Figure S5C

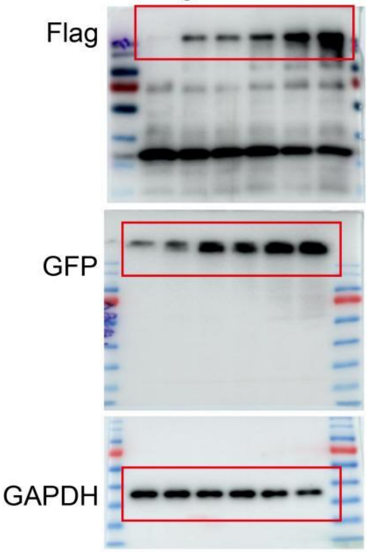

Full unedited gel for Figure S5D

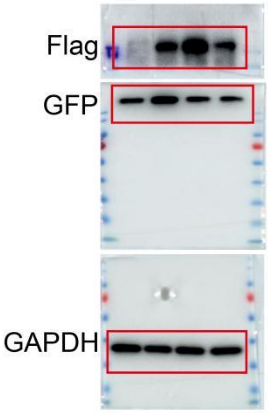

Full unedited gel for Figure S5G

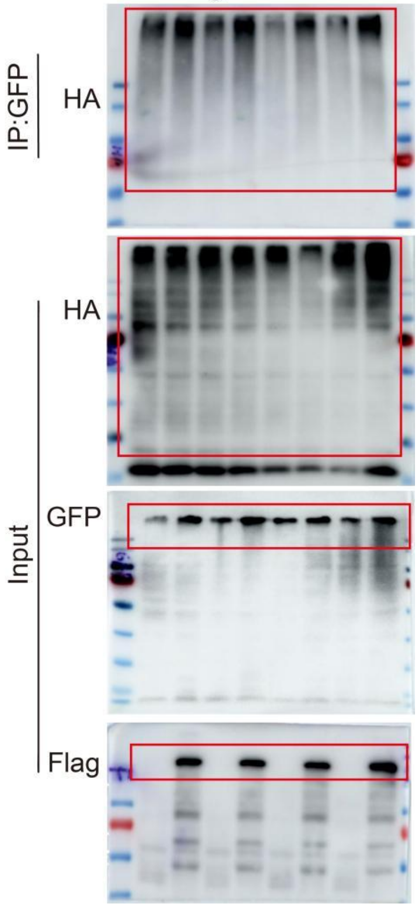

Full unedited gel for Figure S6I

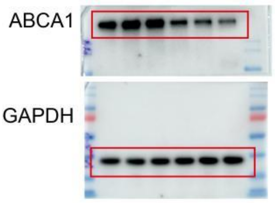

Full unedited gel for Figure S9D

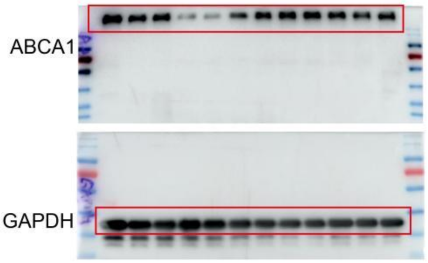

Full unedited gel for Figure S9H

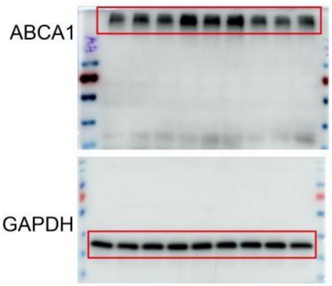

Supplement: Unedited blot and gel images [file jci-135-186509-s079.pdf]
